# Association of the C-reactive protein-albumin-lymphocyte index with clinical outcomes after mechanical thrombectomy for acute ischemic stroke

**DOI:** 10.3389/fnagi.2026.1817455

**Published:** 2026-04-28

**Authors:** Jin Li, Shaohua Qing, Longyi Zheng, Shuang Tang, Jia Duan, Wenli Xing, Ao Qian

**Affiliations:** 1 Department of Cerebrovascular Disease, Suining Central Hospital, Suining, Sichuan, China; 2 Department of Neurology, Suining Central Hospital, Suining, Sichuan, China; 3 Department of Radiology, Suining Central Hospital, Suining, Sichuan, China

**Keywords:** acute ischemic stroke, CALLY index, functional outcome, hemorrhagic transformation, malignant cerebral edema, mechanical thrombectomy, mortality

## Abstract

**Objective:**

This study aims to explore the associations of C-reactive protein-albumin-lymphocyte (CALLY) index with hemorrhagic transformation (HT), malignant cerebral edema (MCE), and 90-day unfavorable outcome and all-cause mortality in patients with acute ischemic stroke (AIS) following mechanical thrombectomy (MT).

**Methods:**

A retrospective study was conducted. The CALLY index was calculated as albumin × lymphocyte count/(CRP × 10). Unfavorable outcome was defined as the modified Rankin Scale score >2 at 90-day follow-up. Multivariable logistic analyses were performed to evaluate the associations between the CALLY index and clinical outcomes. The restricted cubic spines (RCS) curves were employed for pinpointing any nonlinear associations. In addition, mediation analysis was conducted to assess the potential mediating roles of HT and MCE linking the relationship between the CALLY index and 90-day outcomes.

**Results:**

A total of 477 patients were enrolled, demonstrating the rates of HT, MCE, unfavorable outcome, and all-cause mortality were 29.8% (142), 22.9 (109), 54.5 (260), and 25.6 (122), respectively. Multivariable logistic analysis revealed that the CALLY index was associated with reduced risk of HT (odds ratio [OR] 0.843, 95% confidence interval [CI] 0.769–0.924, *p* < 0.001), MCE (OR 0.894, 95% CI 0.824–0.971, *p* = 0.008), unfavorable outcome (OR 0.895, 95% CI 0.840–0.954, *p* = 0.001), and mortality (OR 0.907, 95% CI 0.836–0.984, *p* = 0.019). RCS curves exhibited non-linear relationship between the CALLY index and HT (*p*-non-linear = 0.008), manifesting that CALLY index was associated with HT when its value below 2.681 (OR 0.530, 95% CI 0.357–0.785, *p* = 0.002). Mediation analysis indicated that the association between the CALLY index and unfavorable outcome may be partially mediated by HT (29.1%, *p* < 0.001) and MCE (28.1%, *p* = 0.003).

**Conclusion:**

The CALLY index is associated with reduced risk of HT, MCE, and 90-day unfavorable outcome and all-cause mortality. HT and MCE may be the mediators linking the CALLY index to 90-day unfavorable outcome.

## 1 Introduction

Acute ischemic stroke (AIS) secondary to acute large artery occlusion (LAO) is a catastrophic cerebrovascular condition characterized by high risk of disability and mortality (Zhang et al., 2023). Severe hypoxia of cerebral tissue would be induced by LAO, leading to irreversible progression of ischemic penumbra to infarct core, if timely reperfusion fails to be achieved (Qian et al., 2024). In recent years, progress in neurointerventional technologies has established mechanical thrombectomy (MT) as the standard care for acute LAO in anterior circulation, demonstrating superior clinical outcomes compared to medical management alone (Berkhemer et al., 2015; Goyal et al., 2015). However, complications following MT not only hinder neurological improvement, but may also compromise patient survival. Hemorrhagic transformation (HT) and cerebral edema are common early post-MT complications. Severe HT and malignant cerebral edema (MCE) may lead to marked elevation of intracranial pressure, frequently progressing to life-threatening herniation (Huang et al., 2019). Therefore, identification of factors related to HT and MCE following MT holds potential to enhance risk stratification, and improve prognosis in patients with acute LAO.

Inflammation and nutritional status are critical determinants influencing the prognosis of AIS. The C-reactive protein-albumin-lymphocyte (CALLY) index is a composite marker incorporating C-reactive protein (CRP), albumin, and lymphocyte, quantitatively integrating systemic inflammatory burden and nutritional condition (Chen et al., 2025). The CALLY index was initially used to evaluate prognosis of malignancies, such as hepatocellular carcinoma, gastric cancer, and colorectal cancer (Iida et al., 2022; Zhang et al., 2023; Yang et al., 2023). Recently, the CALLY index has also been applied to evaluate HT and adverse outcome in patients with AIS (Pan et al., 2025). However, few studies have reported the relationship between CALLY index and clinical outcomes in AIS patients treated with MT (Lu et al., 2025; Zhu et al., 2025). Therefore, we conducted this retrospective study with the primary purpose to elucidate the association of CALLY index with post-MT HT and MCE; and secondary objective to evaluate the relationship of CALLY index with 90-day unfavorable outcome and all-cause mortality.

## 2 Methods

### 2.1 Study population

This study has been approved by our institutional ethics committee (Approve number: KYLLKS20250110). All patients treated with MT at Suining Central Hospital from February 2021 to January 2025 were screened. The inclusion criteria were as follows: (1) patients with occlusions in the terminal internal carotid artery (ICA) or M1/M2 segments of middle cerebral artery (MCA); (2) time from symptom or last known well to admission less than 24 h; and (3) age more than 18 years. The exclusion criteria included patients with (1) acute LAO in the posterior circulation; (2) immunodeficiency disease or immunosuppressants use; (3) progressive/decompensated comorbidities, tumors or hematologic disorders at stroke onset; (4) acute or chronic infectious diseases; or (5) miss data of CRP, albumin, lymphocyte at admission, post-MT imaging, and 90-day follow-up; (6) pre-existing disability prior to current AIS onset (modified Rankin Scale [mRS] score > 1). In addition, patients who developed new diseases potentially affecting neurological function within 90-day post-stroke onset were also excluded.

### 2.2 Patient management

All patients with suspected AIS underwent non-contrast head computed tomography (CT) scan immediately upon admission to rule out intracranial hemorrhage, followed by CT angiography to identify the site of arterial occlusion. The intravenous thrombolysis (IVT) with alteplase was performed for patients presenting within 4.5 h of symptom onset after excluding contraindications. Patients with symptoms in the time window of 6–24 h were evaluated through CT perfusion to quantify ischemic penumbra and infarct core. The MT was performed for patients with contraindication or failure of IVT, or obvious penumbra/core mismatch on CTP (penumbra volume ≥15 mL; mismatch ratio ≥1.8) (Chung et al., 2022). All MT procedures were performed under general anesthesia, using either direct aspiration, stent-retriever thrombectomy, or a combined approach. The decision to deploy stents or balloon angioplasty was made at the discretion of surgeons based on arterial stenosis or dissection. All patients were transferred to the neurological intensive care unit (NICU) for postoperative scrutiny. Protocolized dual-energy head CT scans were performed immediately, 24 h, and 48 h after MT. Additional emergent CT scan was also obtained when neurological deterioration was indicated. Post-procedural laboratory measurements were ordered at the discretion of physicians based on clinical judgement.

### 2.3 Data collection

Blood samples were collected for complete blood count, CRP, hepatic and renal function indicators, coagulation profile, and glucose within 30 min of admission. The National Institutes of Health Stroke Scale (NIHSS) was used to assess neurological deficits. The stroke burden was evaluated on the preoperative non-contrast head CT according to the standard Alberta Stroke Program Early CT Score (ASPECTS) (Pexman et al., 2001). The hyperdense middle cerebral artery sign (HMCAS) was defined as a high-density signal observed along the MCA trace on preoperative non-contrast head CT, with MCA attenuation > 43 Hounsfield unit and >1.2 times of normal contralateral vascular density (Koo et al., 2000). Stroke etiology was classified according to the Trail of ORG 10172 in Acute Stroke Treatment (TOAST) criteria, specifically categorizing patients in this study as large-artery atherosclerosis (LAA), cardioembolic, and undermined or other thrombi (Adams et al., 1993). Collateral circulation was assessed based on digital subtraction angiography (DSA) at the initial stage of MT procedure, using the following criteria: grade 0: collateral supply filled less than 1/3 of the occluded arterial territory; grade 1: partial collateral perfusion occupied 1/3–2/3 of the occluded region; and grade 2: collaterals were more than 2/3 of the occluded territory (Christoforidis et al., 2005). Successful recanalization was considered as achieving the final modified Thrombolysis in Cerebral Infarction (mTICI) score of 2b or 3 (Zaidat et al., 2013). Onset to recanalization time was calculated from symptom onset or last known well to successful recanalization of occluded artery or abortion of procedure if recanalization was not achieved.

The data of comorbidity were obtained through patients/family member interviews, medical records, or verification of current medications. Smoking was classified as more than one cigarette use per day, and alcoholism was defined as daily consumption of at least 80 grams of liquor (Sun et al., 2017; Chen et al., 2023).

### 2.4 Clinical outcome and inflammatory index

The primary endpoint was post-MT HT and MCE occurred within 48 h. The diagnosis of HT was based on European-Australasian Acute Stroke Study (ECASS) II criteria, which included both hemorrhagic infarction (HI1 and HI2) and parenchymal hematoma (PH1 and PH2) (Hacke et al., 1998). MCE was defined as an increase in the NIHSS score of ≥4, or ≥1 in the consciousness score, combined with any of the following imaging criteria: (1) hypodensity involving more than 50% of the MCA territory, accompanied by signs of local mass effect, such as lateral ventricle compression and sulcal effacement; (2) midline shift more than 5 mm at the level of the septum pellucidum or pineal gland, with effacement of the basal cistern; or (3) brain swelling involving more than one-third of the hemisphere with obvious midline shift (Huang et al., 2019; Zhang et al., 2023; Zeng et al., 2022; Wiacek et al., 2022). The secondary outcomes aimed to evaluate the 90-day unfavorable outcome and all-cause mortality that assessed by qualified personnel or physicians, who were blinded to the initial CALLY value, through in-person or telephone interviews using the mRS. Specifically, the unfavorable outcome was defined as mRS score > 2, and mortality was regarded as mRS score = 6. The initial CALLY index was calculated as albumin × lymphocyte count/(CRP × 10) for all patients with indicators measured at admission. Furthermore, CALLY index at the first 24 h post MT was also calculated for patients whose relative data were available.

### 2.5 Statistical analysis

Variable selection was informed by previous studies and our clinical experience (Qian et al., 2024, 2025). This study only included patients with complete data of CRP, albumin, lymphocyte, and clinical outcomes. Variables with missing data exceeding 20% were excluded from analysis, while missing value in retained variables were imputed via multiple imputation using the “mice” package in R software. Five imputed datasets were generated with 10 iterations. The imputation model included all variables (Supplementary Table S1) and clinical outcomes. For the main multivariable regression analyses, results were pooled across the five imputed datasets using Rubin' rules (Rubin, 1987). Given the extremely low proportion of the missing data, the five imputed datasets were highly similar (Supplementary Table S2). Therefore, the univariate analysis, restricted cubic spline (RCS) curves, subgroup analysis, sensitivity analysis, and mediation analysis were performed using the first imputed dataset. All continuous variables were non-normally distributed, confirmed by Shapiro–Wilk test, and were presented as median (interquartile range, IQR) that analyzed through Mann–Whitney *U*-test or Kruskal–Wallis H test. Categorical variables were described as number (percentage), and compared with Chi-square test. The receiver operator characteristic (ROC) curves, quantified by area under the curve (AUC), were employed to assess the discriminatory ability of the CALLY index for clinical outcomes. Patients were stratified into quartiles based on the value of CALLY index, with the lowest quartile (Q1) serving as the reference. We established regression models separately for the four clinical outcomes. Sex, age, and variables with statistical significance in univariate analysis were enrolled into models. To mitigate the risk of overfitting, we calculated the maximum number of co-variates for each outcome based on criterion that events-per-variable (EPV) should be at least 10 (Peduzzi et al., 1996). For models with candidate co-variates exceeding the EPV limit, we prioritized variables for inclusion according to statistical significance sorted by *p* value in univariate analysis, and variables with the smallest *p* value were sequentially entered into models until the EPV criterion was reached, while always retaining sex and age. Multicollinearity within the models was assessed using variance inflation factors (VIF), applying a threshold of VIF > 5 to indicate significant collinearity. For established models, internal validation was performed using bootstrapping approach with 1,000 resamples to estimate the optimism-corrected *C*-statistic. Multivariable logistic analyses, adjusting for co-variates in the regression models, were performed to quantitatively assess the associations between CALLY index and clinical outcomes, measured as odds ratio (OR) and 95% confidence interval (CI). The median CALLY index value within each quartile was also analyzed as a continuous variable for linear trend test. The Benjamini–Hochberg false discovery rate (FDR) correction was applied for multiple comparisons to reduce the Type 1 error. RCS curves were used for evaluating any non-linear relationships between the CALLY index and clinical outcomes after adjusting co-variates in the regression models. Planned subgroup analyses were performed through stratifying variables including sex, age (>70 or ≤ 70 years), intravenous thrombolysis, occlusion vessel (ICA or MCA), TOAST classification (LAA or cardioembolism), onset to recanalization time (>350 or ≤ 350 min), and collateral score (grade 0, 1, and 2). Interaction effects between the CALLY index and above stratification variables were tested using likelihood ratio test. Moreover, the discriminatory ability of the CALLY index and other common composite inflammatory indices, including systemic immune-inflammation index (SII, neutrophil count × platelet count/lymphocyte count), systemic inflammation response index (SIRI, neutrophil count × monocyte count/lymphocyte count), neutrophil-to-lymphocyte ratio (NLR), lymphocyte-to-monocyte ratio (LMR), prognostic nutritional index (PNI, albumin + 5 × lymphocyte count), and pan-immune-inflammation value (PIV, neutrophil count × platelet count × monocyte count/lymphocyte count), for clinical outcomes was compared using Delong test. Furthermore, sensitivity analyses were conducted to evaluate the association between CALLY index and clinical outcomes in patients with successful recanalization (mTICI ≥ 2b). In addition, we incorporated the CALLY index into baseline regression models to quantify the incremental discriminatory value through changes in C statistics, the category-free net-reclassification index (NRI), and integrated discrimination improvement (IDI). Finally, we performed mediation analysis to evaluate HT and MCE as potential mediators linking the CALLY index to 90-day outcomes, using bootstrapping approach with 5,000 resamples to estimate the proportion of mediation. All analyses were performed with R software (version 4.4.2, R Foundation for Statistical Computing, https://www.R-project.org/). A two-tailed *p* value < 0.05 was considered as statistical significance.

## 3 Results

### 3.1 Baseline characteristics

**Figure 1:**
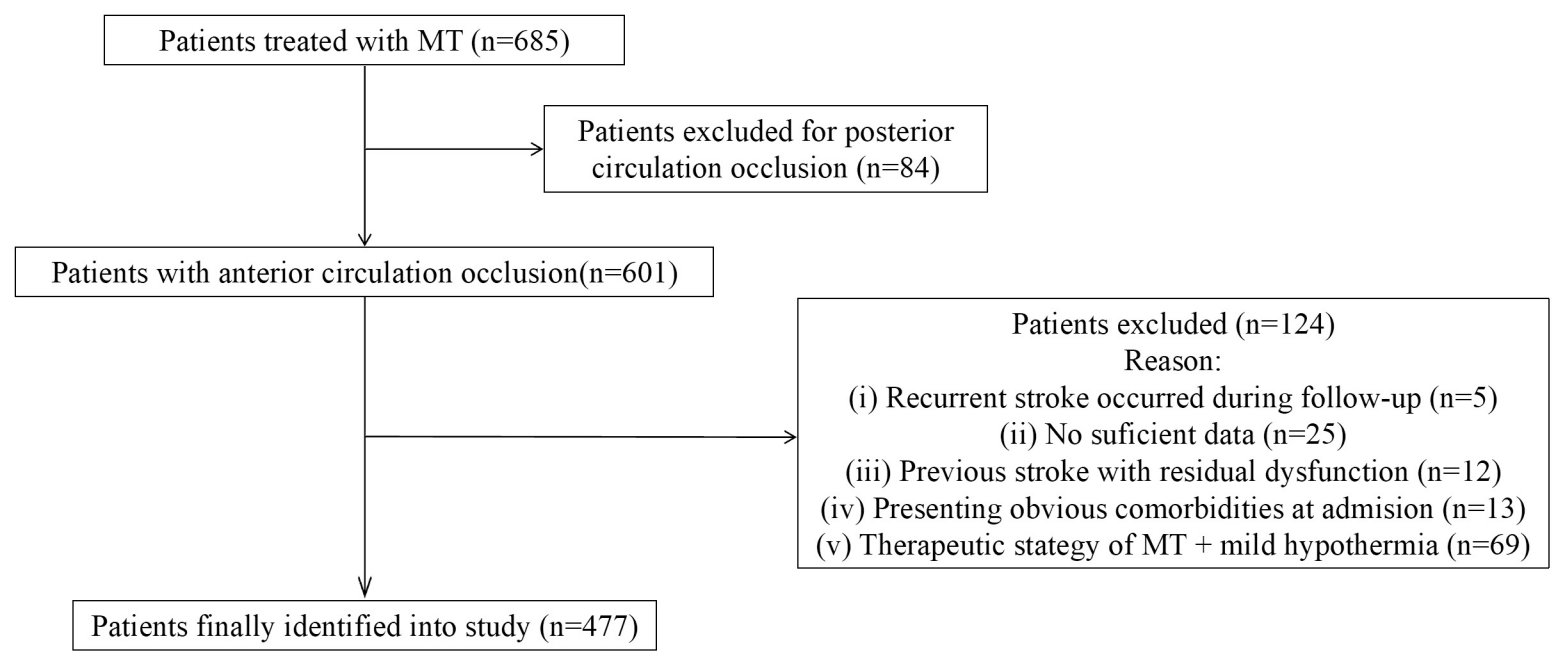
Process of patient selection.

**Figure 2:**
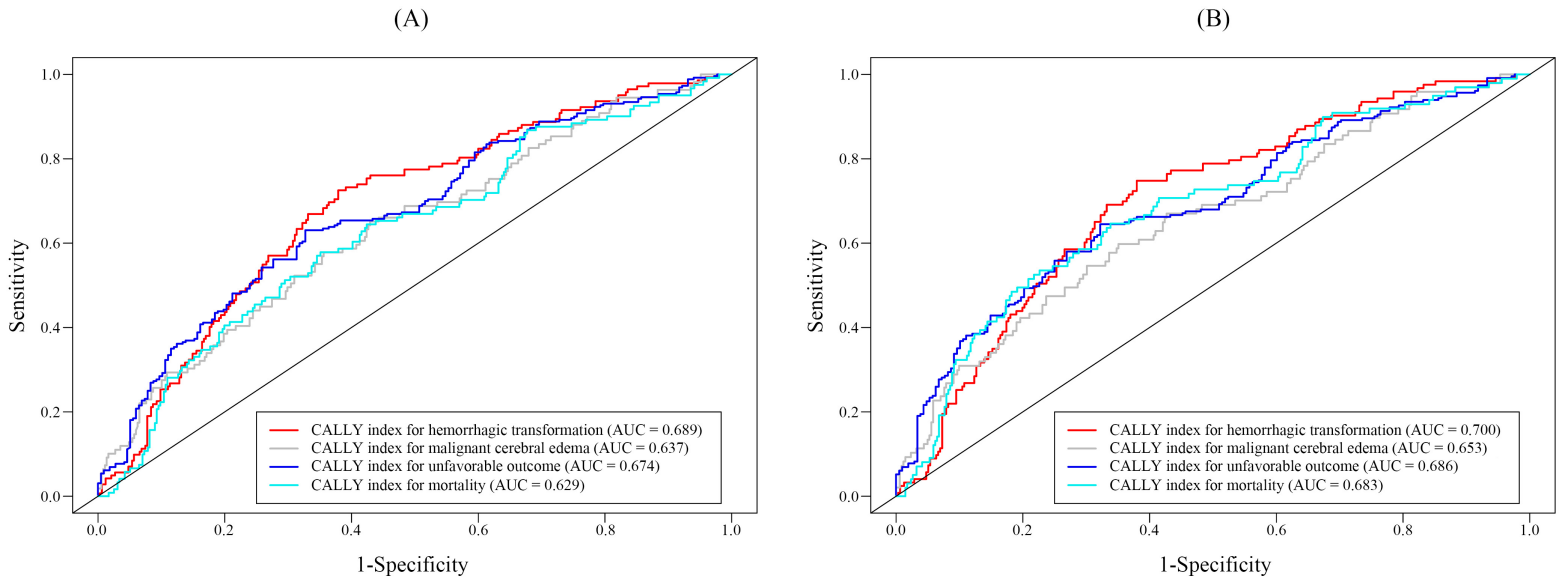
ROC curves showed moderate discriminatory ability of the CALLY index for clinical outcomes. **(A)** All patients (AUC: 0.629–0.689); **(B)** patients with successful recanalization (AUC: 0.653–0.700).

A total of 477 patients were finally enrolled into this study, and the selection process was shown in Figure 1. The baseline characteristics of the patients were presented in Table 1. The value of the CALLY index of these patients was 1.27 (IQR 0.40–3.66). HT occurred in 142 patients (29.8%), and 109 (22.9%) patients experienced MCE. For 90-day outcomes, the rates of unfavorable outcome and mortality were 54.5% (*n* = 260) and 25.6% (*n* = 122), respectively. However, the ROC curves showed only moderate discriminatory ability of CALLY index to clinical outcomes (AUC from 0.629 to 0.689; Figure 2A).

**Table 1:** Baseline characteristics of patients with and without hemorrhagic transformation.

Variables	All	Hemorrhagic transformation	Non-hemorrhagic transformation	*p* value
Patients, *n* (%)	477	142 (29.8)	335 (70.2)	-
Demongraphics				
Female, *n* (%)	212 (44.4)	71 (50.0)	141 (42.1)	0.112
Age (years)	72 [63–78]	73 [67–79]	72 [61–78]	0.092
Smoker, *n* (%)	93 (19.5)	31 (21.8)	62 (18.5)	0.402
Alcoholism, *n* (%)	86 (18.0)	23 (16.2)	63 (18.8)	0.498
Medical history, *n* (%)				
Hypertension	229 (48.0)	67 (47.2)	162 (48.4)	0.814
Diabetes mellitus	98 (20.5)	32 (22.5)	66 (19.7)	0.484
Coronary heart disease	62 (13.0)	18 (12.7)	44 (13.1)	0.892
Atrial fibrillation	247 (51.8)	76 (53.5)	171 (51.0)	0.621
Rheumatic heart disease	35 (7.3)	15 (10.6)	20 (6.0)	0.079
Heart failure	29 (6.1)	8 (5.6)	21 (6.3)	0.791
Prior stroke	63 (13.2)	21 (14.8)	42 (12.5)	0.507
Antiplatelet at onset	24 (5.0)	9 (6.3)	15 (4.5)	0.395
Anticoagulant at onset	47 (9.9)	12 (8.5)	35 (10.4)	0.503
Clinical and imaging characteristics				
Systolic pressure (mmHg)	144 [125–162]	145 [128–164]	144 [124–161]	0.519
Diastolic pressure (mmHg)	83 [75–94]	85 [77–95]	83 [74–94]	0.198
Intravenous thrombolysis, *n* (%)	167 (35.0)	65 (45.8)	102 (30.4)	**0.001**
Initial NIHSS score	15 [11–19]	17 [13–21]	14 [10–18]	**< 0.001**
Baseline ASPECTS	8 [7–9]	8 [7–9]	8 [8–9]	**< 0.001**
Present HMCAS, *n* (%)	167 (35.0)	78 (54.9)	89 (26.6)	**< 0.001**
Occluded vessel region, *n* (%)				
ICA	170 (35.6)	66 (46.5)	104 (31.0)	**0.001**
MCA	307 (64.4)	76 (53.5)	231 (69.0)	
TOAST classification, *n* (%)				
LAA	169 (35.4)	50 (35.2)	119 (35.5)	0.989
Cardioembolic	270 (56.6)	81 (57.0)	189 (56.4)	
Undetermined or others	38 (8.0)	11 (7.7)	27 (8.1)	
Collateral score, *n* (%)				
Grade 0	122 (25.6)	55 (38.7)	67 (20.0)	**< 0.001**
Grade 1	191 (40.0)	45 (31.7)	146 (43.6)	
Grade 2	164 (34.4)	42 (29.6)	122 (36.4)	
Onset to recanalization time (min)	355 [283–449]	390 [317–495]	342 [273–421]	**< 0.001**
Stent implantation, *n* (%)	112 (23.5)	28 (19.7)	84 (25.1)	0.207
Successful recanalization, *n* (%)	439 (92.0)	123 (86.6)	316 (94.3)	0.004
Laboratory findings				
Total calcium (mmol/L)	2.23 [2.14–2.32]	2.23 [2.13–2.31]	2.23 [2.14–2.32]	0.905
Sodium (mmol/L)	139.00 [137.00–140.90]	139.00 [136.47–140.72]	139.00 [137.10–141.00]	0.428
Potassium (mmol/L)	3.80 [3.50–4.07]	3.80 [3.44–4.05]	3.85 [3.52–4.08]	0.234
Chloride (mmol/L)	104.50 [102.60–106.70]	104.80 [102.77–106.72]	104.50 [102.40–106.70]	0.558
Glucose (mmol/L)	7.40 [6.20–9.20]	8.40 [6.80–10.40]	7.00 [6.10–8.80]	**< 0.001**
CRP (mg/L)	3.90 [1.26–11.43]	7.12 [2.65–15.55]	3.04 [1.08–8.55]	**< 0.001**
White blood cell, × 10^9^/L	8.70 [7.20–10.70]	9.10 [7.50–11.52]	8.60 [7.10–10.60]	0.084
Neutrophil, × 10^9^/L	6.98 [5.16–8.92]	7.40 [5.80–9.80]	6.64 [5.01–8.58]	**0.001**
Monocyte, × 10^9^/L	0.46 [0.32–0.61]	0.45 [0.28–0.56]	0.47 [0.32–0.63]	0.111
Lymphocyte, × 10^9^/L	1.18 [0.82–1.65]	0.90 [0.65–1.42]	1.27 [0.89–1.81]	**< 0.001**
Hemoglobin, × 10^9^/L	126 [115–138]	126 [113–138]	126 [116–138]	0.516
Platelet, × 10^9^/L	156 [114–198]	175 [118–210]	149 [114–189]	**0.007**
PT (s)	12.00 [11.10–13.00]	12.20 [11.20–13.10]	12.00 [11.10–12.90]	0.262
TT (s)	17.20 [16.10–18.10]	17.40 [16.27–18.40]	17.10 [16.10–18.10]	0.101
APTT (s)	28.30 [25.70–32.15]	28.15 [25.30–32.62]	28.30 [26.00–32.00]	0.973
Fibrinogen (g/L)	2.92 [2.40–3.39]	2.88 [2.37–3.16]	2.94 [2.45–3.46]	0.177
INR	1.01 [0.95–1.09]	1.02 [0.95–1.10]	1.01 [0.95–1.08]	0.478
Albumin (g/L)	39.50 [36.90–42.05]	39.70 [36.82–42.10]	39.50 [36.90–42.00]	0.692
TC (mmol/L)	4.36 [3.74–5.01]	4.43 [3.73–4.97]	4.34 [3.76–5.07]	0.859
TG (mmol/L)	1.19 [0.88–1.78]	1.22 [0.90–1.95]	1.19 [0.87–1.76]	0.383
LDL-C (mmol/L)	2.17 [1.64–2.65]	2.11 [1.57–2.56]	2.18 [1.68–2.67]	0.417
HDL-C (mmol/L)	1.32 [1.10–1.50]	1.30 [1.05–1.48]	1.33 [1.12–1.53]	0.221
CALLY index	1.27 [0.40–3.66]	0.58 [0.19–1.37]	1.80 [0.61–4.65]	**< 0.001**

Bold values indicate statistical significance.

NIHSS, National Institute of Health stroke scale; ASPECTS, Alberta stroke program early CT score; HMCAS, hyperdense middle cerebral artery sign; ICA, internal carotid artery; MCA, middle cerebral artery; TOAST, trail of ORG 10172 in acute stroke treatment; LAA, large-artery atherosclerosis; CRP, C-reactive protein; PT, prothrombin time; TT, thrombin time; APTT, activated partial thromboplastin time; INR, international normalized ratio; TC, total cholesterol; TG, triglyceride; HDL-C, high-density lipoprotein cholesterol; LDL-C, low-density lipoprotein cholesterol; CALLY index, CRP-albumin-lymphocyte index.

### 3.2 Association between CALLY index and hemorrhagic transformation

**Figure 3:**
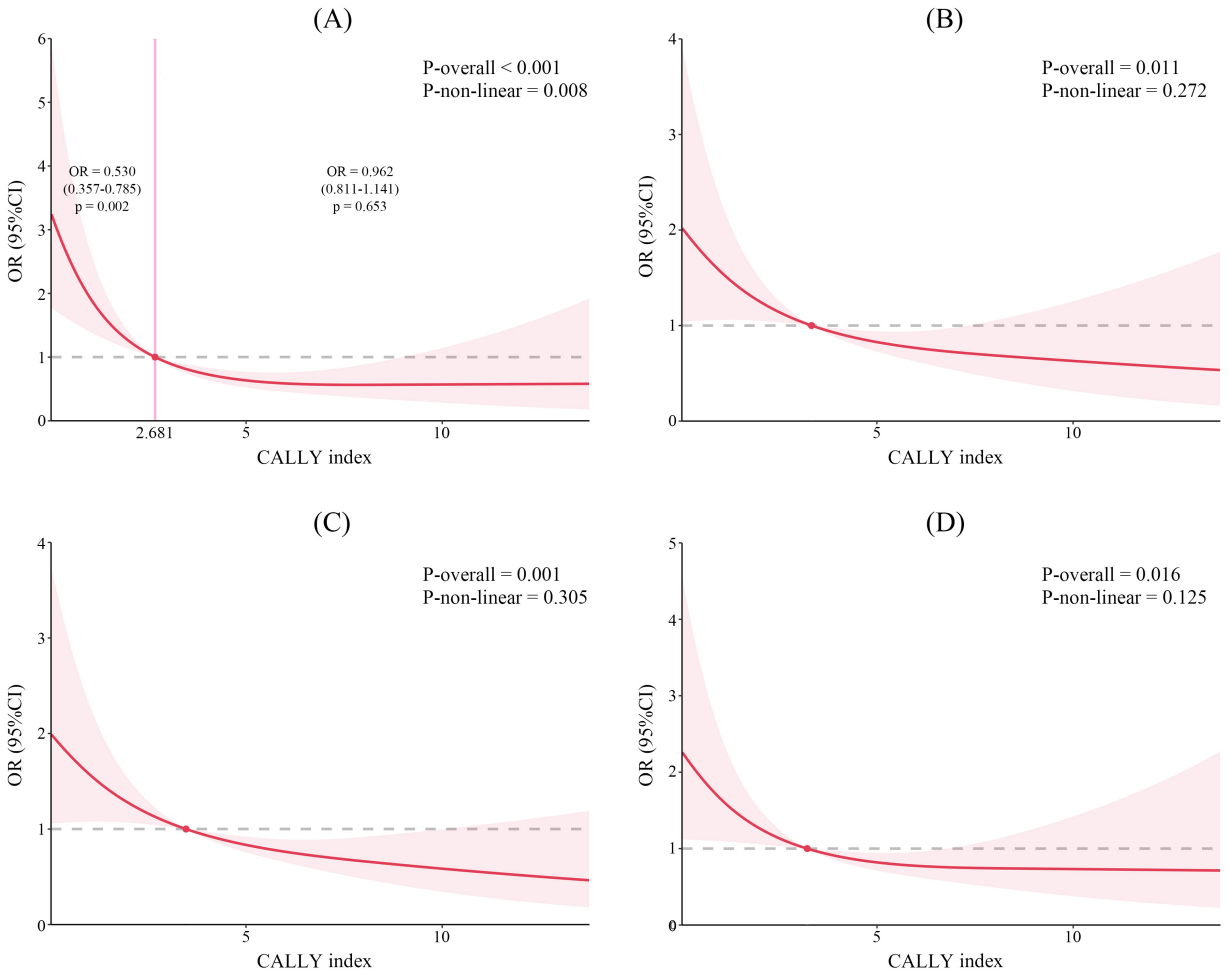
Restricted cubic spline for the association between the CALLY index and clinical outcomes. **(A)** hemorrhagic transformation; **(B)** malignant cerebral edema; **(C)** unfavorable outcome; **(D)** mortality.

**Figure 4:**
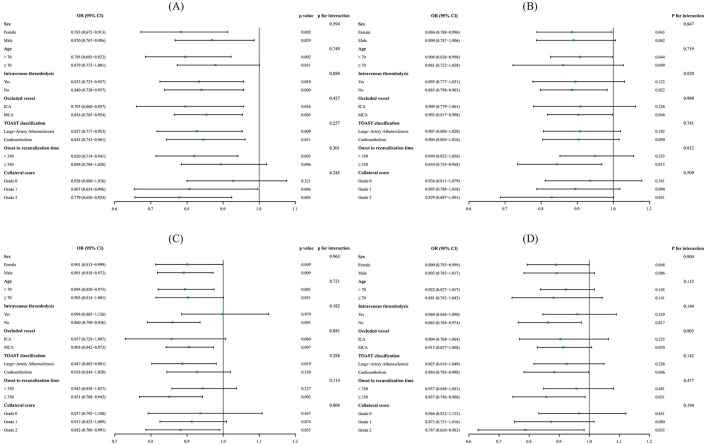
Subgroup analysis revealing the associations between the CALLY index and clinical outcomes under different conditions. **(A)** Hemorrhagic transformation; **(B)** malignant cerebral edema; **(C)** unfavorable outcome; **(D)** mortality.

The baseline characteristics between patients with and without HT were presented in Table 1. Compared with patients without HT, those with HT had higher proportions of IVT, HMCAS, and ICA occlusion. These patients possessed higher initial NIHSS score, lower baseline ASPECTS, worse collateral status, and longer time of onset to recanalization (all *p* < 0.05). Internal validation suggested acceptable overfitting and internal validity (Supplementary Table S3). When analyzed as a continuous variable, the increased CALLY index was significantly associated with decreased risk of HT (OR 0.843, 95% CI 0.769–0.924, *p* < 0.001), after adjusting for sex, age, IVT, initial NIHSS score, baseline ASPECTS, present HMCAS, occluded vessel region, collateral score, onset to recanalization time, successful recanalization, neutrophil, platelet, and glucose (Table 2). No significant multicollinearity was found with all VIF values < 2. When categorizing the CALLY index as an ordinal variable, patients in the highest quartile also shown lowest risk of HT (OR 0.196, 95% CI 0.095–0.403, *p* < 0.001), compared to those in the lowest quartile. After FDR correction, the CALLY index was still associated with HT (continuous: *p*-FDR = 0.001; Q4 vs. Q1, *p*-FDR < 0.001; Supplementary Table S4). Considering that PH2 is the most sever subtype of HT, we also evaluated the relationship between the CALLY index and PH2. Fifty patients (10.4%) have developed PH2, and multivariable regression analysis revealed that the CALLY index remained significantly associated with PH2 (OR 0.801, 95% CI 0.669–0.960, *p* = 0.016). RCS analysis revealed a nonlinear relationship between the CALLY index and HT risk, implying that lower CALLY index was significantly associated with increased HT risk when level below the threshold of 2.681 (OR 0.530, 95% CI 0.357–0.785, *p* = 0.002, *p*-non-linear = 0.008; Figure 3A). Among the whole cohort, 328 patients had CALLY levels below this threshold, and the incidence of HT was 36.5% (*n* = 120), compared to 14.8% (22/149) in patients with CALLY index ≥ 2.681 (*p* < 0.001). Subgroup analysis revealed significant associations between the CALLY index and HT in most subgroups, excepting patients with age ≤ 70 years, onset to recanalization time ≤ 350 min, and collateral score of grade 0 (Figure 4A). No significant interaction effect was found between the CALLY index and these stratification variables (all *p* > 0.05).

**Table 2:** Unadjusted and adjusted results of the associations between CALLY index and outcomes.

Clinical outcomes	Hemorrhagic transformation^a^		Malignant cerebral edema^b^		Unfavorable outcome^c^		Mortality^d^	
	OR (95% CI)	*p* value	OR (95% CI)	*p* value	OR (95% CI)	*p* value	OR (95% CI)	*p* value
Crude								
Continuous	0.807 (0.738–0.883)	< 0.001	0.873 (0.804–0.947)	0.001	0.864 (0.817–0.915)	< 0.001	0.877 (0.812–0.947)	0.001
Quartile								
Q1	Reference		Reference		Reference		Reference	
Q2	0.654 (0.391–1.094)	0.105	0.563 (0.321–0.987)	0.045	0.440 (0.251–0.773)	0.004	0.481 (0.278–0.831)	0.009
Q3	0.239 (0.133–0.429)	< 0.001	0.379 (0.208–0.691)	0.002	0.252 (0.144–0.442)	< 0.001	0.405 (0.231–0.711)	0.002
Q4	0.163 (0.086–0.309)	< 0.001	0.275 (0.144–0.524)	< 0.001	0.154 (0.087–0.273)	< 0.001	0.184 (0.095–0.358)	< 0.001
*P* for trend		< 0.001		0.001		< 0.001		< 0.001
Adjusted								
Continuous	0.843 (0.769–0.924)	< 0.001	0.894 (0.824–0.971)	0.008	0.895 (0.840–0.954)	0.001	0.907 (0.836–0.984)	0.019
Quartile								
Q1	Reference		Reference		Reference		Reference	
Q2	0.646 (0.355–1.176)	0.153	0.442 (0.237–0.825)	0.010	0.443 (0.224–0.873)	0.019	0.442 (0.225–0.870)	0.018
Q3	0.294 (0.150–0.575)	< 0.001	0.435 (0.226–0.837)	0.013	0.397 (0.203–0.774)	0.007	0.565 (0.284–1.122)	0.103
Q4	0.196 (0.095–0.403)	< 0.001	0.299 (0.149–0.602)	0.001	0.233 (0.118–0.460)	< 0.001	0.211 (0.095–0.466)	< 0.001
*P* for trend		< 0.001		0.008		< 0.001		0.001

^a^Adjusted for sex, age, intravenous thrombolysis, initial NIHSS score, baseline ASPECTS, present HMCAS, occluded vessel region, collateral score, onset to recanalization time, successful recanalization, neutrophil, platelet, and glucose.

^b^Adjusted for sex, age, history of hypertension, initial NIHSS score, baseline ASPECTS, present HMCAS, collateral score, platelet, and glucose.

^c^Adjusted for sex, age, history of hypertension, initial NIHSS score, baseline ASPECTS, present HMCAS, occluded vessel region, collateral score, onset to recanalization time, successful recanalization, total calcium, sodium, neutrophil, platelet, glucose, PT, TG, and HDL-C.

^d^Adjusted for sex, age, systolic pressure, initial NIHSS score, baseline ASPECTS, present HMCAS, occluded vessel region, collateral score, successful recanalization, platelet, and glucose.

NIHSS, National Institute of Health stroke scale; ASPECTS, Alberta stroke program early CT score; HMCAS, hyperdense middle cerebral artery sign; PT, prothrombin time; TG, triglyceride; HDL-C, high-density lipoprotein cholesterol.

### 3.3 Association between CALLY index and malignant cerebral edema

Patients with MCE were older, had higher initial NIHSS score, and were more likely to have a history of hypertension, presence of HMCAS, and ICA occlusion, compared to those without MCE. Moreover, these patients exhibited lower ASPECTS and more adverse collateral score (all *p* < 0.05; Supplementary Table S5). After adjusting for sex, age, history of hypertension, initial NIHSS score, baseline ASPECTS, presence of HMCAS, collateral score, platelet, and glucose, we identified a significant association between CALLY index, whether treated as continuous (OR 0.894, 95% CI 0.824–0.971, *p* = 0.008) or ordinal variable (Q4 vs. Q1: OR 0.299, 95% CI 0.149–0.602, *p* = 0.001), and MCE (Table 2). The RCS curve suggested that an elevated CALLY index was linearly associated with a reduced risk of MCE (*p*-non-linear = 0.272; Figure 3B). Subgroup analysis indicated no significant interaction between the CALLY index and the stratification variables (all *p* for interaction > 0.05). However, significant associations with MCE were only observed in female patients, and those with age >70 years, no IVT therapy, MCA occlusion, and onset to recanalization time ≤ 350 min (Figure 4B).

### 3.4 Association between CALLY index and 90-day unfavorable outcome

Compare to patients with favorable outcome, those experiencing unfavorable outcome were significantly older, and possessed higher rates of hypertension history, elevated initial NIHSS score, and lower baseline ASPECTS. Moreover, these patients exhibited higher prevalence of HMCAS, ICA occlusion, prolonged onset to recanalization time, and poorer collateral circulation, and less incidence of successful recanalization (all *p* < 0.05; Supplementary Table S6). Multivariable logistic analysis, with adjustment of sex, age, history of hypertension, initial NIHSS score, baseline ASPECTS, present HMCAS, occluded vessel region, collateral score, onset to recanalization time, successful recanalization, total calcium, sodium, neutrophil, platelet, glucose, PT, TG, and HDL-C, demonstrated that both higher value (OR 0.895, 95% CI 0.840–0.954, *p* = 0.001) and highest level (Q4 vs. Q1: OR 0.233, 95% CI 0.118–0.460, *p* < 0.001) of CALLY index were associated with decreased risk of 90-day unfavorable outcome (Table 2). The RCS curve indicated that the CALLY index exhibited non-linear relationship with reduced unfavorable outcome (*p*-non-linear = 0.305; Figure 3C). Subgroup analyses revealed the robust relationship of CALLY index and unfavorable outcome in male, and patients with age >70 years, no IVT, MCA occlusion, TOAST classification of LAA, time of onset to recanalization ≤ 350 min, and collateral score of grade 2 (all *p* < 0.05; Figure 4C). The CALLY index showed no significant interaction with stratification variables (*p* for interaction > 0.05).

### 3.5 The association between CALLY index and 90-day all-cause mortality

Compared with 90-day survivors, non-survivors were older and more frequently female. They exhibited higher admission systolic blood pressure and initial NIHSS score, and presented with higher prevalence of HMCAS and ICA occlusion. Moreover, these patients had lower baseline ASPECTS and poorer collateral circulation (all *p* < 0.05; Supplementary Table S7). In multivariable logistic regression analysis that adjusted for sex, age, systolic pressure, initial NIHSS score, baseline ASPECTS, presence of HMCAS, occluded vessel region, collateral score, successful recanalization, platelet, and glucose, the CALLY index showed a significant association with mortality risk when treated as a continuous variable (OR 0.907, 95% CI 0.836–0.984, *p* = 0.019). In the analysis of the CALLY index as a categorical variable, patients in the highest quartile had a significantly lower risk of mortality compared to those in the lowest quartile (Q4 vs. Q1: OR 0.211, 95% CI 0.095–0.466, *p* < 0.001; Table 2). The RCS curve indicated that the CALLY index was also linearly associated with reduced risk of mortality (*p*-non-linear = 0.125; Figure 3D). Subgroup analysis revealed that associations between CALLY index and mortality remained significant in female patients, and those with no IVT therapy, cardioembolism, onset to recanalization time ≤ 350 min, and collateral score of grade 2 (all *p* < 0.05; Figure 4D). No significant interaction was also observed between CALLY index and stratification variables (all *p* for interaction >0.05).

### 3.6 Discriminatory ability comparison of the CALLY index with other composite inflammatory indices for clinical outcomes

We evaluated the discriminatory ability of the CALLY index and six composite inflammatory indices, including SII, SIRI, NLR, LMR, PNI, and PIV, for clinical outcomes by comparing AUC values (Supplementary Table S8). We found that the CALLY index showed the highest AUC value for HT. While, no significant difference of AUC values was observed between the CALLY index and other markers for MCE. For unfavorable outcome, the CALLY index had significantly higher AUC value than LMR (*p* = 0.048) and PNI (*p* = 0.013). However, for mortality, the AUC of the CALLY index was significantly lower than that of SII (*p* = 0.013).

### 3.7 Sensitivity analysis revealing the relationship between the CALLY index and clinical outcomes in patients with successful recanalization

To validate the robustness of the associations between the CALLY index and clinical outcomes, we performed a sensitivity analysis in patients with successful recanalization (mTICI ≥ 2b). A total of 439 patients achieved successful recanalization, demonstrating that the rates of HT, MCE, and 90-day unfavorable outcome and mortality were 28.0% (*n* = 123), 22.1% (*n* = 97), 52.6% (*n* = 231), and 22.5 (*n* = 99), respectively. ROC curves indicated that the discriminatory ability of the CALLY index for these four outcomes, as measured by AUC, ranged from 0.653 to 0.700 (Figure 2B). After adjusting for the aforementioned co-variates in the regression models, we found that the CALLY index also significantly associated with reduced risks of HT (continuous: OR 0.807, 95% CI 0.719–0.905, *p* < 0.001; Q4 vs. Q1: OR 0.151, 95% CI 0.066–0.345, *p* < 0.001), MCE (continuous: OR 0.873, 95% CI 0.795–0.959, *p* = 0.005; Q4 vs. Q1: OR 0.238, 95% CI 0.112–0.509, *p* < 0.001), unfavorable outcome (continuous: OR 0.888, 95% CI 0.830–0.951, *p* = 0.001; Q4 vs. Q1: OR 0.177, 95% CI 0.086–0.366, *p* < 0.001), and mortality (continuous: OR 0.877, 95% CI 0.795–0.968, *p* = 0.009; Q4 vs. Q1: OR 0.141, 95% CI 0.059–0.338, *p* < 0.001; Table 3).

**Table 3:** Associations between CALLY index and outcomes in patients with successful recanalization.

Clinical outcomes	Hemorrhagic transformation^a^		Malignant cerebral edema^b^		Unfavorable outcome^c^		Mortality^d^	
	OR (95% CI)	*p* value	OR (95% CI)	*p* value	OR (95% CI)	*p* value	OR (95% CI)	*p* value
Crude								
Continuous	0.770 (0.690–0.859)	< 0.001	0.853 (0.777–0.937)	0.001	0.858 (0.807–0.912)	< 0.001	0.832 (0.753–0.919)	< 0.001
Q1	Reference		Reference		Reference		Reference	
Q2	0.591 (0.344–1.015)	0.056	0.470 (0.259–0.852)	0.013	0.341 (0.188–0.619)	< 0.001	0.323 (0.177–0.589)	< 0.001
Q3	0.227 (0.122–0.422)	< 0.001	0.355 (0.190–0.666)	0.001	0.193 (0.106–0.350)	< 0.001	0.290 (0.157–0.536)	< 0.001
Q4	0.132 (0.065–0.267)	< 0.001	0.226 (0.112–0.452)	< 0.001	0.129 (0.070–0.236)	< 0.001	0.115 (0.053–0.251)	< 0.001
*P* for trend		< 0.001		< 0.001		< 0.001		< 0.001
Adjusted								
Continuous	0.807 (0.719–0.905)	< 0.001	0.873 (0.795–0.959)	0.005	0.888 (0.830–0.951)	0.001	0.877 (0.795–0.968)	0.009
Q1	Reference		Reference		Reference		Reference	
Q2	0.604 (0.318–1.149)	0.124	0.393 (0.203–0.760)	0.006	0.319 (0.156–0.651)	0.002	0.327 (0.163–0.655)	0.002
Q3	0.298 (0.144–0.616)	0.001	0.421 (0.213–0.835)	0.013	0.275 (0.136–0.556)	< 0.001	0.411 (0.202–0.836)	0.014
Q4	0.151 (0.066–0.345)	< 0.001	0.238 (0.112–0.509)	< 0.001	0.177 (0.086–0.366)	< 0.001	0.141 (0.059–0.338)	< 0.001
*P* for trend		< 0.001		0.003		< 0.001		< 0.001

^a^Adjusted for sex, age, intravenous thrombolysis, initial NIHSS score, baseline ASPECTS, present HMCAS, occluded vessel region, collateral score, onset to recanalization time, neutrophil, platelet, glucose.

^b^Adjusted for sex, age, history of hypertension, initial NIHSS score, baseline ASPECTS, present HMCAS, collateral score, platelet, glucose.

^c^Adjusted for sex, age, history of hypertension, initial NIHSS score, baseline ASPECTS, present HMCAS, occluded vessel region, collateral score, onset to recanalization time, total calcium, sodium, neutrophil, platelet, glucose, PT, TG, HDL-C,

^d^Adjusted for sex, age, systolic pressure, initial NIHSS score, baseline ASPECTS, present HMCAS, occluded vessel region, collateral score, platelet, glucose.

NIHSS, National Institute of Health stroke scale; ASPECTS, Alberta stroke program early CT score; HMCAS, hyperdense middle cerebral artery sign; PT, prothrombin time; TG, triglyceride; HDL-C: high-density lipoprotein cholesterol.

### 3.8 Association between post mechanical thrombectomy CALLY index and clinical outcomes

Recognizing that MT may provoke profound inflammatory and stress response, which would substantially confound the interpretation of the baseline CALLY index, we also collected data on the CALLY index within 24 h following the procedure. Meanwhile, to avoid bias from HT and MCE that may already manifested at the time of the assessment, we only evaluated the associations between the post-MT CALLY index and 90-day outcomes. The post-MT CALLY index was available in 351 patients [0.24 (IQR 0.13–0.46)]. The baseline characteristics of patients with and without post-MT CALLY index were summarized in SupplementaryTable S9. After adjusting for co-variates, the associations were still significant between the CALLY index and the reduced risk of unfavorable outcome (continuous: OR 0.588, 95% CI 0.351–0.986, *p* = 0.044; Q4 vs. Q1: OR 0.175, 95% CI 0.076–0.402, *p* < 0.001) and mortality (continuous: OR 0.305, 95% CI 0.109–0.852, *p* = 0.024; Q4 vs. Q1: OR 0.199, 95% CI 0.083–0.477, *p* < 0.001; Table 4).

**Table 4:** Associations between post mechanical thrombectomy CALLY index and 90-day outcomes.

Clinical outcomes	Unfavorable outcome^a^		Mortality^b^	
	OR (95% CI)	*p* value	OR (95% CI)	*p* value
Crude				
Continuous	0.523 (0.316–0.865)	0.012	0.299 (0.116–0.772)	0.013
Q1	Reference		Reference	
Q2	0.335 (0.173–0.648)	0.001	0.442 (0.225–0.870)	0.018
Q3	0.222 (0.115–0.430)	< 0.001	0.298 (0.143–0.620)	0.001
Q4	0.185 (0.095–0.358)	< 0.001	0.246 (0.114–0.529)	< 0.001
*P* for trend		< 0.001		0.001
Adjusted				
Continuous	0.588 (0.351–0.986)	0.044	0.305 (0.109–0.852)	0.024
Q1	Reference		Reference	
Q2	0.281 (0.123–0.642)	0.003	0.245 (0.108–0.557)	0.001
Q3	0.196 (0.086–0.444)	< 0.001	0.198 (0.083–0.470)	< 0.001
Q4	0.175 (0.076–0.402)	< 0.001	0.199 (0.083–0.477)	< 0.001
*P* for trend		0.001		0.004

^a^Adjusted for sex, age, history of hypertension, initial NIHSS score, baseline ASPECTS, present HMCAS, occluded vessel region, collateral score, onset to recanalization time, successful recanalization, total calcium, sodium, neutrophil, platelet, glucose, PT, TG, HDL-C.

^b^Adjusted for sex, age, systolic pressure, initial NIHSS score, baseline ASPECTS, present HMCAS, occluded vessel region, collateral score, successful recanalization, platelet, glucose.

### 3.9 Incremental prognostic value of the CALLY index

To investigate the clinical utility of the CALLY index, we incorporated it into the regression models to evaluate the incremental prognostic value. The results were summarized in Table 5. We found that adding the CALLY index into the models significantly improved the discriminatory ability for HT and unfavorable outcome, as demonstrated by increments in the *C*-statistic, category-free NRI, and IDI (all *p* < 0.05). In the analysis of MCE, inclusion the CALLY index modestly enhances the *C*-statistic (from 0.738 to 0.749, *p* = 0.294), while yielding significant IDI (*p* = 0.008) and category-free NRI (*p* = 0.032). For mortality, adding the CALLY index resulted in limited improvement in *C*-statistic (*p* = 0.283) and IDI (*p* = 0.080), while leading to significant increase in category-free NRI (*p* = 0.002).

**Table 5:** Reclassification and discrimination statistics for outcomes by CALLY index.

Models	*C*-statistic		Category-free NRI		IDI	
	Estimate (95% CI)	*p* value	Estimate (95% CI), %	*p* value	Estimate (95% CI), %	*p* value
Hemorrhagic transformation						
Basic model^a^	0.785 (0.741–0.829)	Reference		Reference		Reference
Basic model^a^ + CALLY index	0.806 (0.765–0.848)	0.034	44.5 (27.5–61.5)	< 0.001	3.2 (1.7–4.8)	< 0.001
Malignant cerebral edema						
Basic model^b^	0.738 (0.685–0.790)	Reference		Reference		Reference
Basic model^b^ + CALLY index	0.749 (0.699–0.800)	0.294	20.9 (1.7–40.0)	0.032	1.6 (0.4–2.7)	0.008
Unfavorable outcome						
Basic model^c^	0.826 (0.789–0.862)	Reference		Reference		Reference
Basic model^c^ + CALLY index	0.839 (0.804–0.874)	0.038	38.9 (22.4–55.4)	< 0.001	2.3 (1.0–3.7)	< 0.001
Mortality						
Basic model^d^	0.824 (0.780–0.866)	Reference		Reference		Reference
Basic model^d^ + CALLY index	0.829 (0.787–0.871)	0.283	27.3 (9.3–45.3)	0.002	0.9 (-0.1–1.9)	0.080

^a^Including sex, age, intravenous thrombolysis, initial NIHSS score, baseline ASPECTS, present HMCAS, occluded vessel region, collateral score, onset to recanalization time, successful recanalization, neutrophil, platelet, glucose.

^b^Including sex, age, history of hypertension, initial NIHSS score, baseline ASPECTS, present HMCAS, collateral score, platelet, glucose.

^c^Including sex, age, history of hypertension, initial NIHSS score, baseline ASPECTS, present HMCAS, occluded vessel region, collateral score, onset to recanalization time, successful recanalization, total calcium, sodium, neutrophil, platelet, glucose, PT, TG, HDL-C.

^d^Including sex, age, systolic pressure, initial NIHSS score, baseline ASPECTS, present HMCAS, occluded vessel region, collateral score, successful recanalization, platelet, glucose.

NIHSS, National Institute of Health stroke scale; ASPECTS, Alberta stroke program early CT score; HMCAS, hyperdense middle cerebral artery sign; PT, prothrombin time; TG, triglyceride; HDL-C, high-density lipoprotein cholesterol.

CALLY index: CRP-albumin-lymphocyte index; NRI, net-reclassification index; IDI, integrated discrimination improvement.

### 3.10 Relationships among the CALLY index, early complications, and 90-day outcomes

**Figure 5:**
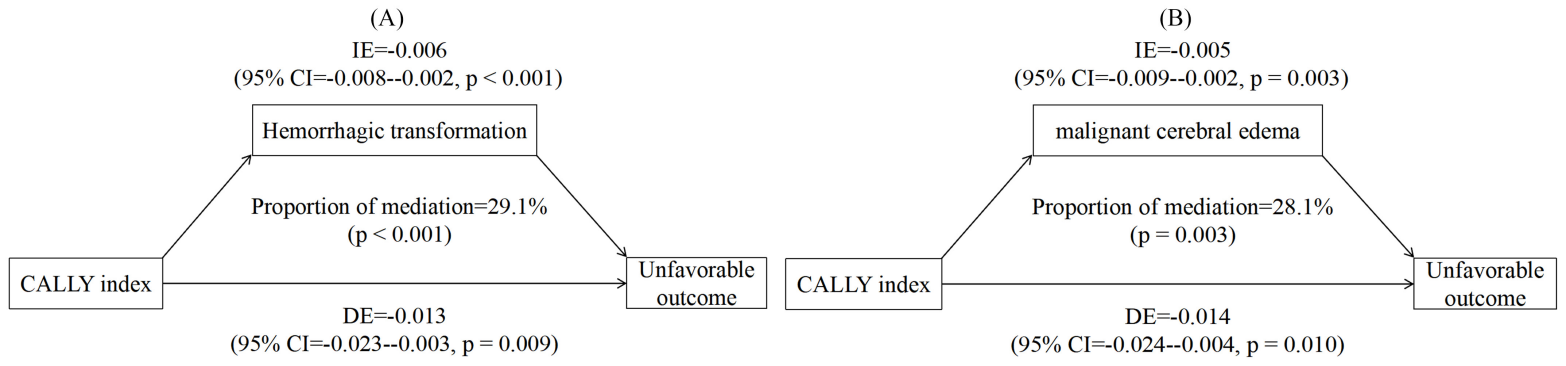
Mediation analysis indicating the mediating role of hemorrhagic transformation **(A)** and malignant cerebral edema **(B)** linking the CALLY index and 90-day unfavorable outcome.

On the basis of the relationships between the CALLY index and clinical outcomes described above, we further explored whether early complications (HT and MCE) served as mediators linking the CALLY index measured at admission to 90-day outcomes. Mediation analysis revealed that the effect of the CALLY index on 90-day unfavorable outcome was partially mediated by HT and MCE, with mediation proportions of 29.1% (*p* < 0.001) and 28.1% (*p* = 0.003), respectively (Figure 5).

## 4 Discussion

In this study, we investigated the associations between admission CALLY index and clinical outcomes following MT for acute LAO in anterior circulation. The CALLY index showed significant correlations with HT, MCE, and 90-day unfavorable outcome and all-cause mortality, even after adjustment for potential confounders. Similar associations were also identified in patients with successful recanalization and post-MT CALLY index. Furthermore, incorporating the CALLY index could enhanced the discriminatory capacity of most models. Therefore, these findings suggested that the CALLY index may be a potential marker for prognostic evaluation in patients with acute LAO in anterior circulation following MT.

The CALLY index, comprehensively reflecting inflammatory, immune, and nutritional status, was first proposed in 2021 for prognostic assessment in cancer patients (Yi et al., 2021). Recently, with the role of inflammatory-immune response in the development and progression of AIS attracting increasing attention (Ji et al., 2022), several studies have also explored the relationship between the CALLY index and clinical prognosis in patients with AIS. (Pan et al. 2025) reported significant associations between the CALLY index and both HT and poor functional outcomes in stroke patients receiving medical treatment. Similarly, (Lu et al. 2025) confirmed the relationship between reduced CALLY index levels and increased adverse outcome in AIS patients undergoing endovascular therapy. Consistent with these observations, our study also confirmed that elevated CALLY index levels correlated with reduced risk of HT and unfavorable outcome. Extension of current evidence further identified this relationship not only with early complications (HT and MCE), but also with 90-day outcomes. These findings contribute to our comprehensive evaluation of the prognostic value of the CALLY index in patients with AIS following MT.

Separately explaining the biological roles of the CALLY index constituents could elucidate the mechanisms for the association with outcomes in AIS. As an acute inflammatory biomarker, CRP actively contributes to local cerebral and systemic inflammation in AIS. CRP concentration potentially reflects the systemic load and functional state of proinflammatory cytokines. Patients with elevated CRP may exhibit heightened susceptibility to exaggerated inflammation response following stroke that may correlate with an increased propensity for adverse outcomes (Di Napoli et al., 2001; Luan and Yao, 2018). Clinically, elevated CRP is also identified as an independent factor of early neurologic deterioration and poor prognosis in patients with AIS (Seo et al., 2012). Lymphocyte may serve as a primary neuroprotective role through suppression of inflammatory cascades and attenuation of infiltrating immune cell recruitment and activation (Cao et al., 2024). However, following AIS, under stress condition, the hypothalamic-pituitary-adrenal axis elevates catecholamine and cortisol levels, triggering apoptosis and functional suppression of peripheral lymphocytes (Lattanzi et al., 2021). Albumin, a multifunctional plasma protein synthesized in the liver, regulates plasma oncotic pressure, facilitates molecular transports, and modulates inflammatory response (Roche et al., 2008). However, accumulating clinical evidence indicates that the efficacy of albumin therapy in patients with ischemic stroke is suboptimal. In the part 1 of the ALIAS trail, researchers failed to find superiority of albumin treatment for achieving good neurological outcomes in ischemic stroke patients (albumin group vs. control group = 37.8% vs. 36.9%) (Hill et al., 2011). Furthermore, in the part 2 of the ALIAS trail, the results also suggested no clinical benefit of albumin therapy in patients with ischemic stroke (Ginsberg et al., 2013). Nevertheless, increased clinical observations put forward the association of hypoalbuminemia and adverse post-stroke outcomes (Morotti et al., 2017; Ko et al., 2022). Thus, future researches are still required to explore the role of albumin in stroke patients.

In this study, we confirmed that the CALLY index was associated with both common early complication (HT and MCE) and late outcome (90-day unfavorable outcome and mortality) following MT. The RCS analysis revealed a nonlinear relationship between the CALLY index and HT, suggesting an exploratory threshold effect that the association of higher CALLY level with lower HT risk appeared more pronounced when the CALLY value was below 2.681. However, this threshold value may be sample-specific and requires external validation. A potential explanation may be the distinct pathophysiology of HT, which involves acute BBB disruption and reperfusion injuring that may require a critical level of systemically inflammatory and nutritional balance, as reflected by the CALLY index in this study, to be effectively mitigated. In contrast, the linear relationship with MCE, unfavorable outcome, and mortality may reflect the cumulative impact of systemic inflammation on sustained neuroinflammatory processes, cerebral edema progression, and overall prognosis.

In recent years, numerous factors associated with prognosis after MT have been proposed. Therefore, we compared the discriminatory ability of the CALLY index and six common composite inflammatory indices for clinical outcomes to evaluate the prognostic value of the CALLY index within the inflammatory markers. For HT, MCE, and unfavorable outcome, the CALLY index demonstrated discriminatory ability that was superior or comparable with other composite inflammatory indices. However, for mortality, the CALLY index had lower discriminatory capacity than SII. As a biomarker integrating neutrophil, platelet, and lymphocyte, SII may play a critical role of inflammation-immune response in adverse outcomes among patients with acute LAO, which has also been confirmed in our previous study (Qian et al., 2024). Comparisons of CALLY index and other composite inflammatory indices in this study was not to compete with existing inflammatory markers, but to explore whether a marker integrating both inflammatory and nutritional components could offer complementary value in evaluating post-MT outcomes. Despite the discriminatory ability inferior to SII for most clinical outcomes, the CALLY index may underscore the important role of nutritional status in stroke prognosis, providing a novel perspective for patient management and mechanical investigation.

Although the CALLY index demonstrated significant associations with multiple clinical outcomes, the utility as a standalone prognostic tool was constrained by the moderate discriminatory ability. We observed that adding the CALLY index to established models significantly improved risk reclassification for HT and unfavorable outcome, while improvement in model discrimination of MCE and mortality was modest. These findings suggested that the incremental value of the CALLY index was manifested in refining risk classification, which more accurately identifies patients at high risk. It is also appropriate to regard the CALLY index as a supplementary factor within multifactorial risk assessment framework, rather than as an independent prognostic biomarker.

Considering that the CALLY index is easily disturbed, directly attributing the effect on long-term prognosis may lack robustness. Therefore, as a hypothesis-generating analysis, we explored whether early complications (HT and MCE) mediated the association between the CALLY index and 90-day outcomes. The mediation analysis suggested that the association between a lower CALLY index and 90-day unfavorable outcome may be partially mediated by HT and MCE. However, no significant mediation was observed for mortality. This phenomenon may be explained by the distinct nature of these outcomes. Functional recovery is closely linked to the extent of early brain injury and complications, such as HT and MCE, which could directly impair neurological rehabilitation (Qian et al., 2025). In contrast, mortality represents a more complex, multifactorial endpoint influenced by a broader range of systemic factors that may dilute the mediating role of early complications (Qian et al., 2024). It is also possible that the CALLY index influences survival through pathways independent of HT and MCE, a hypothesis that warrants further investigation. Critically, several methodological caveats should be considered when interpreting these mediation results. Mediation analysis in observational studies can only identify statistical pathways, rather establish biological or causal mediation. Thus, these findings should be viewed as hypothesis-generating that underscore the requirement of future prospective studies to elucidate the underlying mechanisms linking the CALLY index to post-stroke outcomes.

Certainly, this study has several limitations. First, its retrospective single-center design may constrain the generalizability of the findings. Second, our analysis assessed the CALLY index at only two time points, precluding evaluation of the dynamic trajectory and relationship with clinical outcomes. Third, despite adjusting for a wide range of co-variates, the possibility of residual confounding from unmeasured or unrecorded factors cannot be fully excluded, such as number of thrombectomy passes, specific procedural technique, medication use, detailed nutritional status, peri-procedural blood pressure management, other complications, like infection, and rehabilitation interventions, all of which may influence both systemic inflammation and risk of adverse outcomes. The unavailability of these data precluded us from fully disentangling the independent effect of the CALLY index. Therefore, although our study provided robust evidence of associations, the findings should be interpreted with caution. Fourth, the discriminatory capacity of the CALLY index for clinical outcomes, reflected by the AUC value, was modest, suggesting that the CALLY index should not be interpreted as a standalone tool for evaluating prognosis. The clinical utility of the CALLY index may be reflected by the improvement of risk reclassification. Fifth, the variable selection strategy for regression models was partially data-driven that co-variates were prioritized based on univariate *p* value. Given the exploratory nature of this study and the limited number of events for some outcomes, we employed this approach while taking active steps to mitigate overfitting that enforcing an EPV ≥ 10 criterion to restrict the number of co-variates and performing bootstrap interval validation. Nevertheless, variables selection based on the same dataset still introduce optimism in model performance. Thus, external validation is essential to confirm the generalizability of our findings. Sixth, the post-MT CALLY index data were available for only 351 of 477 patients, because post procedure measurements of CRP, albumin, and lymphocyte count were not mandatory, and were obtained at the discretion of physicians. Critically, the analysis of post-MT CALLY index was not intended to be representative of the entire cohort, but to explore the relationship between post-MT CALLY index and long-term outcomes in a real-world clinical setting where such testing is mainly guided by clinical judgment. Consequently, the subgroup analysis based on post-MT CALLY index are subject to selection bias, as the significant difference in the baseline characteristics and outcomes. The direction of this bias likely overestimates the true associations that patients with post-MT CALLY data have lower rate of mortality. We suggest that these results should be interpreted as exploratory, and future prospective studies with protocol of post-MT measurements are needed to validate our observations. Furthermore, early complications (HT and MCE) occurring within 24 h could potentially influence post-MT CALLY values, while we were unable to determine whether the post-MT laboratory measurements were obtained before or after the occurrence of these early complications. Therefore, we cannot explore the relationships between post-MT CALLY index and early complications. Seventh, the sample size of our cohort was modest. Several subgroup analyses were based on relatively small sample size, resulting in wide CIs and limited statistical precision, indicating that these findings should be interpreted as exploratory, and caution is warranted when drawing conclusions from subgroups with small number of cases. Finally, as an observational study, our findings demonstrated statistical associations between the identified factors and clinical outcomes, rather than prediction or causation.

## 5 Conclusion

The CALLY index is associated with reduced risk of HT, MCE, unfavorable outcome, and mortality for patients with acute LAO in anterior circulation following MT. These relationships are also found in patients with successful recanalization and post-MT data. HT and MCE appeared to partially mediate the correlation between the CALLY index and 90-day unfavorable outcome, whereas further studies are required to explore the mechanisms linking the CALLY index to mortality.

## Data Availability

The raw data supporting the conclusions of this article will be made available by the authors, without undue reservation.

## Supplementary material

Supplementary Table S1 Summary of missing data.

Supplementary Table S2 Comparisons of variables with missing values among complete and imputed datasets.

Supplementary Table S3 Internal validation of regression models for clinical outcomes.

Supplementary Table S4 False discovery rate correction for multiple comparisons.

Supplementary Table S5 Baseline characteristics of patients with and without malignant cerebral edema.

Supplementary Table S6 Baseline characteristics of patients with 90-day unfavorable and favorable outcome.

Supplementary Table S7 Baseline characteristics of patients with and without 90-day mortality.

Supplementary Table S8 Comparisons between the CALLY index and other composite inflammatory indices using Delong tests.

Supplementary Table S9 Baseline characteristics of patients with and without post-MT CALLY index.
