# Supplementary material for: Association of the C-reactive protein-albumin-lymphocyte index with clinical outcomes after mechanical thrombectomy for acute ischemic stroke
Source: Front Aging Neurosci. 2026 Apr 28;18:1817455. doi: 10.3389/fnagi.2026.1817455 (PMC13161055; doi:10.3389/fnagi.2026.1817455)
Supplement: Supplementary Table S1 — Summary of missing data. [file Data_Sheet_1.docx]

Supplementary Table S1. Summary of missing data.

| Variables | Missing proportion | Included into study | Comparison between imputed data and complete data |
| --- | --- | --- | --- |
| Sex | 0 | Yes | - |
| Age | 0 | Yes | - |
| Smoker | 0 | Yes | - |
| Alcoholism | 0 | Yes | - |
| Hypertension | 0 | Yes | - |
| Diabetes mellitus | 0 | Yes | - |
| Coronary heart disease | 0 | Yes | - |
| Atrial fibrillation | 0 | Yes | - |
| Rheumatic heart disease | 0 | Yes | - |
| Heart failure | 0 | Yes | - |
| Prior stroke | 0 | Yes | - |
| Antiplatelet at onset | 0 | Yes | - |
| Anticoagulant at onset | 0 | Yes | - |
| Systolic pressure | 0 | Yes | - |
| Diastolic pressure | 0 | Yes | - |
| Intravenous thrombolysis | 0 | Yes | - |
| Initial NIHSS score | 0 | Yes | - |
| Baseline ASPECTS | 0 | Yes | - |
| Present HMCAS | 0 | Yes | - |
| Occluded vessel region | 0 | Yes | - |
| TOAST classification | 0 | Yes | - |
| Collateral score | 0 | Yes | - |
| Onset to recanalization time | 0 | Yes | - |
| Stent implantation | 0 | Yes | - |
| Successful recanalization | 0 | Yes | - |
| Total calcium | 2.1 | Yes | 2.23 [2.14-2.32] vs. 2.23 [2.14-2.32], p = 0.971 |
| Sodium | 0 | Yes | - |
| Potassium | 0 | Yes | - |
| Chloride | 0 | Yes | - |
| Glucose | 0 | Yes | - |
| CRP | 0 | Yes | - |
| White blood cell | 0 | Yes | - |
| Neutrophil | 0 | Yes | - |
| Monocyte | 0 | Yes | - |
| Lymphocyte | 0 | Yes | - |
| Hemoglobin | 0 | Yes | - |
| Platelet | 0 | Yes | - |
| PT | 0 | Yes | - |
| TT | 0 | Yes | - |
| APTT | 0 | Yes | - |
| Fibrinogen | 0 | Yes | - |
| INR | 0 | Yes | - |
| Albumin | 0 | Yes | - |
| TC | 2.1 | Yes | 4.36 [3.74-5.01] vs. 4.36 [3.74-5.01], p = 0.994 |
| TG | 2.1 | Yes | 1.19 [0.88-1.78] vs. 1.19 [0.88-1.78], p = 0.946 |
| LDL-C | 2.1 | Yes | 2.17 [1.64-2.65] vs. 2.18 [1.65-2.65], p = 0.911 |
| HDL-C | 2.1 | Yes | 1.32 [1.10-1.50] vs. 1.32 [1.11-1.50], p = 0.950 |
| Lactic acid | 42.3 | No | - |
| Apo A1 | 29.1 | No | - |
| Apo B | 29.1 | No | - |
| glycated hemoglobin | 41.5 | No | - |
| Number of thrombectomy passes | 42.3 | No | - |
| Thrombectomy techniques | 42.3 | No | - |

NIHSS: National Institute of Health Stroke Scale; ASPECTS: Alberta Stroke Program Early CT Score; HMCAS: Hyperdense middle cerebral artery sign; ICA: Internal carotid artery; MCA: Middle cerebral artery; TOAST: Trail of ORG 10172 in Acute Stroke Treatment; LAA: Large-artery atherosclerosis; CRP: C-reactive protein; PT: Prothrombin time; TT: Thrombin time; APTT: Activated partial thromboplastin time; INR: International normalized ratio; TC: Total cholesterol; TG: Triglyceride; HDL-C: High-density lipoprotein cholesterol; LDL-C: Low-density lipoprotein cholesterol; Apo: apolipoprotein.

Supplementary Table S2. Comparisons of variables with missing values among complete and imputed datasets.

| Variables | Complete dataset | Imputed dataset 1 | Imputed dataset 2 | Imputed dataset 3 | Imputed dataset 4 | Imputed dataset 5 | p value |
| --- | --- | --- | --- | --- | --- | --- | --- |
| Total calcium | 2.23 [2.14-2.32] | 2.23 [2.14-2.32] | 2.23 [2.14-2.32] | 2.23 [2.14-2.32] | 2.23 [2.14-2.32] | 2.22 [2.14-2.32] | 1.000 |
| TC | 4.36 [3.74-5.01] | 4.36 [3.74-5.01] | 4.36 [3.74-5.04] | 4.37 [3.77-5.04] | 4.37 [3.75-5.04] | 4.35 [3.74-5.01] | 1.000 |
| TG | 1.19 [0.88-1.78] | 1.19 [0.88-1.78] | 1.19 [0.88-1.78] | 1.19 [0.88-1.78] | 1.19 [0.88-1.78] | 1.19 [0.88-1.78] | 1.000 |
| LDL-C | 2.18 [1.65-2.65] | 2.17 [1.64-2.65] | 2.18 [1.65-2.65] | 2.18 [1.66-2.65] | 2.18 [1.66-2.65] | 2.18 [1.65-2.65] | 1.000 |
| HDL-C | 1.32 [1.10-1.50] | 1.32 [1.10-1.50] | 1.32 [1.10-1.50] | 1.31 [1.11-1.50] | 1.32 [1.11-1.51] | 1.32 [1.11-1.50] | 1.000 |

TC: Total cholesterol; TG: Triglyceride; HDL-C: High-density lipoprotein cholesterol; LDL-C: Low-density lipoprotein cholesterol

Supplementary Table S3. Internal validation of regression models for clinical outcomes

| Clinical outcomes | Original C-statistic | Optimism-corrected C-statistic^e^ | Slope Optimism | Calibration slope | Calibration intercept |
| --- | --- | --- | --- | --- | --- |
| Hemorrhagic transformation^a^ | 0.785 | 0.758 | 0.1462 | 0.854 | -0.098 |
| Malignant cerebral edema^b^ | 0.738 | 0.709 | 0.1497 | 0.850 | -0.154 |
| Unfavorable outcome^c^ | 0.826 | 0.797 | 0.1552 | 0.845 | 0.013 |
| Mortality^d^ | 0.824 | 0.803 | 0.1047 | 0.895 | -0.096 |

^a^ Adjusted for sex, age, intravenous thrombolysis, initial NIHSS score, baseline ASPECTS, present HMCAS, occluded vessel region, collateral score, onset to recanalization time, successful recanalization, neutrophil, platelet, and glucose.

^b^ Adjusted for sex, age, history of hypertension, initial NIHSS score, baseline ASPECTS, present HMCAS, collateral score, platelet, and glucose.

^c^ Adjusted for sex, age, history of hypertension, initial NIHSS score, baseline ASPECTS, present HMCAS, occluded vessel region, collateral score, onset to recanalization time, successful recanalization, total calcium, sodium, neutrophil, platelet, glucose, PT, TG, and HDL-C.

^d^ Adjusted for sex, age, systolic pressure, initial NIHSS score, baseline ASPECTS, present HMCAS, occluded vessel region, collateral score, successful recanalization, platelet, and glucose.

^e^ Derived from 1000 bootstrap resamples.

NIHSS: National Institute of Health Stroke Scale; ASPECTS: Alberta Stroke Program Early CT Score; HMCAS: Hyperdense middle cerebral artery sign; PT: Prothrombin time; TG: Triglyceride; HDL-C: High-density lipoprotein cholesterol.

Supplementary Table S4. False discovery rate correction for multiple comparisons.

|  | Hemorrhagic transformation^a^ | |  | Malignant cerebral edema^b^ | |  | Unfavorable outcome^c^ | |  | Mortality^d^ | |
| --- | --- | --- | --- | --- | --- | --- | --- | --- | --- | --- | --- |
|  | p value | p-FDR value |  | p value | p-FDR value |  | p value | p-FDR value |  | p value | p-FDR value |
| Continuous | < 0.001 | 0.001 |  | 0.008 | 0.013 |  | 0.001 | 0.002 |  | 0.019 | 0.021 |
| Quartile |  |  |  |  |  |  |  |  |  |  |  |
| Q1 | Reference | |  | Reference | |  | Reference | |  | Reference | |
| Q2 | 0.153 | 0.153 |  | 0.010 | 0.017 |  | 0.019 | 0.022 |  | 0.018 | 0.022 |
| Q3 | < 0.001 | 0.001 |  | 0.013 | 0.019 |  | 0.007 | 0.013 |  | 0.103 | 0.110 |
| Q4 | < 0.001 | < 0.001 |  | 0.001 | 0.002 |  | < 0.001 | < 0.001 |  | < 0.001 | < 0.001 |

^a^ Adjusted for sex, age, intravenous thrombolysis, initial NIHSS score, baseline ASPECTS, present HMCAS, occluded vessel region, collateral score, onset to recanalization time, successful recanalization, neutrophil, platelet, and glucose.

^b^ Adjusted for sex, age, history of hypertension, initial NIHSS score, baseline ASPECTS, present HMCAS, collateral score, platelet, and glucose.

^c^ Adjusted for sex, age, history of hypertension, initial NIHSS score, baseline ASPECTS, present HMCAS, occluded vessel region, collateral score, onset to recanalization time, successful recanalization, total calcium, sodium, neutrophil, platelet, glucose, PT, TG, and HDL-C.

^d^ Adjusted for sex, age, systolic pressure, initial NIHSS score, baseline ASPECTS, present HMCAS, occluded vessel region, collateral score, successful recanalization, platelet, and glucose.

NIHSS: National Institute of Health Stroke Scale; ASPECTS: Alberta Stroke Program Early CT Score; HMCAS: Hyperdense middle cerebral artery sign; PT: Prothrombin time; TG: Triglyceride; HDL-C: High-density lipoprotein cholesterol.

Supplementary Table S5. Baseline characteristics of patients with and without malignant cerebral edema.

|  | Malignant cerebral edema | Non malignant cerebral edema | p value |
| --- | --- | --- | --- |
| **Patients, n (%)** | 109 (22.9) | 368 (77.1) | - |
| **Demongraphics** |  |  |  |
| Female, n (%) | 57 (52.3) | 155 (42.1) | 0.060 |
| Age (years) | 75 [68-81] | 71 [61-78] | **< 0.001** |
| Smoker, n (%) | 18 (16.5) | 75 (20.4) | 0.371 |
| Alcoholism, n (%) | 16 (14.7) | 70 (19.0) | 0.300 |
| **Medical history, n (%)** |  |  |  |
| Hypertension | 67 (61.5) | 162 (44.0) | **0.001** |
| Diabetes mellitus | 20 (18.3) | 78 (21.2) | 0.518 |
| Coronary heart disease | 9 (8.3) | 53 (14.4) | 0.094 |
| Atrial fibrillation | 49 (45.0) | 198 (53.8) | 0.104 |
| Rheumatic heart disease | 8 (7.3) | 27 (7.3) | 0.999 |
| Heart failure | 3 (2.8) | 26 (7.1) | 0.098 |
| Prior stroke | 15 (13.8) | 48 (13.0) | 0.846 |
| Antiplatelet at onset | 4 (3.7) | 20 (5.4) | 0.459 |
| Anticoagulant at onset | 8 (7.3) | 39 (10.6) | 0.316 |
| **Clinical and imaging characteristics** |  |  |  |
| Systolic pressure (mmhg) | 145 [126-170] | 144 [124-160] | 0.204 |
| Diastolic pressure (mmhg) | 83 [74-95] | 83 [75-94] | 0.578 |
| Intravenous thrombolysis, n (%) | 38 (34.9) | 129 (35.1) | 0.971 |
| Initial NIHSS score | 17 [12-22] | 14 [11-18] | **< 0.001** |
| Baseline ASPECTS | 8 [7-9] | 8 [8-9] | **0.002** |
| Present HMCAS, n (%) | 52 (47.7) | 115 (31.3) | **0.002** |
| Occluded vessel region, n (%) |  |  | **0.011** |
| ICA | 50 (45.9) | 120 (32.6) |  |
| MCA | 59 (54.1) | 248 (67.4) |  |
| TOAST classification, n (%) |  |  | 0.409 |
| LAA | 37 (33.9) | 132 (35.9) |  |
| Cardioembolic | 60 (55.0) | 210 (57.1) |  |
| Undetermined or others | 12 (11.1) | 26 (7.0) |  |
| Collateral score, n (%) |  |  | **0.002** |
| Grade 0 | 41 (37.6) | 81 (22.0) |  |
| Grade 1 | 41 (37.6) | 150 (40.8) |  |
| Grade 2 | 27 (24.8) | 137 (37.2) |  |
| Onset to recanalization time (min) | 374 [301-471] | 352 [279-432] | 0.135 |
| Stent implantation, n (%) | 26 (23.9) | 86 (23.4) | 0.917 |
| Successful recanalization, n (%) | 97 (89.0) | 342 (92.9) | 0.182 |
| **Laboratory findings** |  |  |  |
| Total calcium (mmol/L) | 2.24 [2.14-2.32] | 2.22 [2.14-2.32] | 0.316 |
| Sodium (mmol/L) | 139.00 [135.40-140.60] | 139.05 [137.20-140.97] | 0.105 |
| Potassium (mmol/L) | 3.85 [3.53-4.20] | 3.80 [3.50-4.06] | 0.360 |
| Chloride (mmol/L) | 104.20 [102.00-106.55] | 104.50 [102.62-106.70] | 0.754 |
| Glucose (mmol/L) | 8.10 [6.80-10.20] | 7.05 [6.10-8.90] | **< 0.001** |
| CRP (mg/L) | 5.99 [2.05-15.60] | 3.44 [1.16-10.21] | **0.001** |
| White blood cell, × 10^9^/L | 8.60 [7.40-10.75] | 8.75 [7.20-10.70] | 0.466 |
| Neutrophil, × 10^9^/L | 7.23 [5.38-9.36] | 6.83 [5.04-8.86] | 0.091 |
| Monocyte, × 10^9^/L | 0.45 [0.27-0.57] | 0.46 [0.32-0.63] | 0.280 |
| Lymphocyte, × 10^9^/L | 1.04 [0.61-1.43] | 1.23 [0.86-1.74] | **< 0.001** |
| Hemoglobin, × 10^9^/L | 126 [113-139] | 126 [116-138] | 0.726 |
| Platelet, × 10^9^/L | 183 [134-218] | 149 [112-189] | **< 0.001** |
| PT (s) | 12.00 [11.05-13.10] | 12.00 [11.10-13.00] | 0.763 |
| TT (s) | 17.20 [16.30-18.10] | 17.10 [16.00-18.17] | 0.256 |
| APTT (s) | 28.00 [25.30-33.80] | 28.30 [25.90-31.67] | 0.794 |
| Fibrinogen (g/L) | 2.96 [2.39-3.41] | 2.91 [2.42-3.36] | 0.935 |
| INR | 1.02 [0.95-1.08] | 1.01 [0.96-1.09] | 0.569 |
| Albumin (g/L) | 39.50 [35.75-41.85] | 39.50 [37.10-42.07] | 0.322 |
| TC (mmol/L) | 4.52 [3.90-5.23] | 4.33 [3.69-4.99] | 0.104 |
| TG (mmol/L) | 1.28 [0.88-1.99] | 1.18 [0.87-1.72] | 0.189 |
| LDL-C (mmol/L) | 2.19 [1.65-2.76] | 2.16 [1.64-2.62] | 0.517 |
| HDL-C (mmol/L) | 1.29 [1.06-1.50] | 1.33 [1.12-1.50] | 0.201 |
| CALLY index | 0.69 [0.15-2.33] | 1.57 [0.50-4.00] | **< 0.001** |

Bold values indicate statistical significance.

NIHSS: National Institute of Health Stroke Scale; ASPECTS: Alberta Stroke Program Early CT Score; HMCAS: Hyperdense middle cerebral artery sign; ICA: Internal carotid artery; MCA: Middle cerebral artery; TOAST: Trail of ORG 10172 in Acute Stroke Treatment; LAA: Large-artery atherosclerosis; CRP: C-reactive protein; PT: Prothrombin time; TT: Thrombin time; APTT: Activated partial thromboplastin time; INR: International normalized ratio; TC: Total cholesterol; TG: Triglyceride; HDL-C: High-density lipoprotein cholesterol; LDL-C: Low-density lipoprotein cholesterol; CALLY index: CRP-albumin-lymphocyte index.

Supplementary Table S6. Baseline characteristics of patients with 90-day unfavorable and favorable outcome.

|  | Unfavorable outcome | Favorable outcome | p value |
| --- | --- | --- | --- |
| **Patients, n (%)** | 260 (54.5) | 217 (45.5) | - |
| **Demongraphics** |  |  |  |
| Female, n (%) | 126 (48.5) | 86 (39.6) | 0.053 |
| Age (years) | 74 [67-80] | 70 [59-77] | **< 0.001** |
| Smoker, n (%) | 49 (18.8) | 44 (20.3) | 0.695 |
| Alcoholism, n (%) | 42 (16.2) | 44 (20.3) | 0.243 |
| **Medical history, n (%)** |  |  |  |
| Hypertension | 138 (53.1) | 91 (41.9) | **0.015** |
| Diabetes mellitus | 59 (22.7) | 39 (18.0) | 0.204 |
| Coronary heart disease | 36 (13.8) | 26 (12.0) | 0.546 |
| Atrial fibrillation | 140 (53.8) | 107 (49.3) | 0.323 |
| Rheumatic heart disease | 18 (6.9) | 17 (7.8) | 0.704 |
| Heart failure | 11 (4.2) | 18 (8.3) | 0.064 |
| Prior stroke | 39 (15.0) | 24 (11.1) | 0.206 |
| Antiplatelet at onset | 14 (5.4) | 10 (4.6) | 0.699 |
| Anticoagulant at onset | 27 (10.4) | 20 (9.2) | 0.670 |
| **Clinical and imaging characteristics** |  |  |  |
| Systolic pressure (mmhg) | 144 [123-164] | 144 [125-160] | 0.712 |
| Diastolic pressure (mmhg) | 83 [74-93] | 83 [75-94] | 0.580 |
| Intravenous thrombolysis, n (%) | 92 (35.4) | 75 (34.6) | 0.851 |
| Initial NIHSS score | 16 [13-20] | 13 [9-16] | **< 0.001** |
| Baseline ASPECTS | 8 [7-9] | 9 [8-9] | **< 0.001** |
| Present HMCAS, n (%) | 107 (41.2) | 60 (27.6) | **0.002** |
| Occluded vessel region, n (%) |  |  | **0.030** |
| ICA | 104 (40.0) | 66 (30.4) |  |
| MCA | 156 (60.0) | 151 (69.6) |  |
| TOAST classification, n (%) |  |  | 0.388 |
| LAA | 85 (32.7) | 84 (38.7) |  |
| Cardioembolic | 153 (58.8) | 117 (53.9) |  |
| Undetermined or others | 22 (8.5) | 16 (7.4) |  |
| Collateral score, n (%) |  |  | **< 0.001** |
| Grade 0 | 95 (36.5) | 27 (12.4) |  |
| Grade 1 | 95 (36.5) | 96 (44.2) |  |
| Grade 2 | 70 (27.0) | 94 (43.4) |  |
| Onset to recanalization time (min) | 372 [303-473] | 334 [266-420] | **< 0.001** |
| Stent implantation, n (%) | 58 (22.3) | 54 (24.9) | 0.508 |
| Successful recanalization, n (%) | 231 (88.8) | 208 (95.9) | **0.005** |
| **Laboratory findings** |  |  |  |
| Total calcium (mmol/L) | 2.25 [2.15-2.33] | 2.20 [2.12-2.29] | **0.001** |
| Sodium (mmol/L) | 139.00 [136.50-140.60] | 139.20 [137.40-141.35] | **0.013** |
| Potassium (mmol/L) | 3.83 [3.51-4.10] | 3.80 [3.50-4.02] | 0.091 |
| Chloride (mmol/L) | 104.20 [102.32-106.40] | 104.70 [102.80-106.80] | 0.287 |
| Glucose (mmol/L) | 7.90 [6.62-10.01] | 6.80 [5.80-8.49] | **< 0.001** |
| CRP (mg/L) | 5.58 [1.89-14.32] | 2.74 [0.97-6.54] | **< 0.001** |
| White blood cell, × 10^9^/L | 8.85 [7.40-11.07] | 8.60 [6.95-10.35] | **0.025** |
| Neutrophil, × 10^9^/L | 7.33 [5.62-9.56] | 6.45 [4.81-8.20] | **< 0.001** |
| Monocyte, × 10^9^/L | 0.45 [0.29-0.61] | 0.48 [0.32-0.60] | 0.684 |
| Lymphocyte, × 10^9^/L | 1.02 [0.69-1.46] | 1.37 [1.00-1.87] | **< 0.001** |
| Hemoglobin, × 10^9^/L | 125 [114-137] | 127 [116-139] | 0.241 |
| Platelet, × 10^9^/L | 163 [118-206] | 144 [111-183] | **0.002** |
| PT (s) | 12.25 [11.20-13.10] | 11.80 [11.00-12.85] | **0.030** |
| TT (s) | 17.20 [16.10-18.10] | 17.20 [16.10-18.40] | 0.633 |
| APTT (s) | 28.40 [25.82-33.37] | 28.00 [25.60-31.60] | 0.222 |
| Fibrinogen (g/L) | 2.93 [2.48-3.36] | 2.91 [2.38-3.41] | 0.838 |
| INR | 1.02 [0.95-1.09] | 1.01 [0.96-1.08] | 0.333 |
| Albumin (g/L) | 39.50 [36.52-41.60] | 39.60 [37.10-42.30] | 0.229 |
| TC (mmol/L) | 4.35 [3.79-5.06] | 4.37 [3.71-5.00] | 0.964 |
| TG (mmol/L) | 1.32 [0.91-1.97] | 1.11 [0.85-1.59] | **0.004** |
| LDL-C (mmol/L) | 2.18 [1.64-2.73] | 2.16 [1.64-2.58] | 0.368 |
| HDL-C (mmol/L) | 1.26 [1.06-1.45] | 1.36 [1.16-1.58] | **< 0.001** |
| CALLY index | 0.79 [0.23-2.42] | 1.92 [0.83-5.46] | **< 0.001** |

Bold values indicate statistical significance.

NIHSS: National Institute of Health Stroke Scale; ASPECTS: Alberta Stroke Program Early CT Score; HMCAS: Hyperdense middle cerebral artery sign; ICA: Internal carotid artery; MCA: Middle cerebral artery; TOAST: Trail of ORG 10172 in Acute Stroke Treatment; LAA: Large-artery atherosclerosis; CRP: C-reactive protein; PT: Prothrombin time; TT: Thrombin time; APTT: Activated partial thromboplastin time; INR: International normalized ratio; TC: Total cholesterol; TG: Triglyceride; HDL-C: High-density lipoprotein cholesterol; LDL-C: Low-density lipoprotein cholesterol; CALLY index: CRP-albumin-lymphocyte index.

Supplementary Table S7. Baseline characteristics of patients with and without 90-day mortality.

|  | Mortality | Survival | p value |
| --- | --- | --- | --- |
| **Patients, n (%)** | 122 (25.6) | 355 (74.4) | - |
| **Demongraphics** |  |  |  |
| Female, n (%) | 67 (54.9) | 145 (40.8) | **0.007** |
| Age (years) | 76 [69-82] | 71 [61-77] | **< 0.001** |
| Smoker, n (%) | 19 (15.6) | 74 (20.8) | 0.205 |
| Alcoholism, n (%) | 21 (17.2) | 65 (18.3) | 0.786 |
| **Medical history, n (%)** |  |  |  |
| Hypertension | 67 (54.9) | 162 (45.6) | 0.077 |
| Diabetes mellitus | 24 (19.7) | 74 (20.8) | 0.782 |
| Coronary heart disease | 17 (13.9) | 45 (12.7) | 0.721 |
| Atrial fibrillation | 60 (49.2) | 187 (52.7) | 0.505 |
| Rheumatic heart disease | 10 (8.2) | 25 (7.0) | 0.673 |
| Heart failure | 4 (3.3) | 25 (7.0) | 0.133 |
| Prior stroke | 17 (13.9) | 46 (13.0) | 0.783 |
| Antiplatelet at onset | 6 (4.9) | 18 (5.1) | 0.947 |
| Anticoagulant at onset | 10 (8.2) | 37 (10.4) | 0.477 |
| **Clinical and imaging characteristics** |  |  |  |
| Systolic pressure (mmhg) | 151 [135-170] | 144 [124-160] | **0.004** |
| Diastolic pressure (mmhg) | 85 [77-95] | 83 [74-93] | 0.132 |
| Intravenous thrombolysis, n (%) | 45 (36.9) | 122 (34.4) | 0.615 |
| Initial NIHSS score | 18 [14-23] | 14 [10-18] | **< 0.001** |
| Baseline ASPECTS | 8 [7-9] | 8 [8-9] | **< 0.001** |
| Present HMCAS, n (%) | 64 (52.5) | 103 (29.0) | **< 0.001** |
| Occluded vessel region, n (%) |  |  | **< 0.001** |
| ICA | 64 (52.5) | 106 (29.9) |  |
| MCA | 58 (47.5) | 249 (70.1) |  |
| TOAST classification, n (%) |  |  | 0.239 |
| LAA | 37 (30.3) | 132 (37.2) |  |
| Cardioembolic | 72 (59.0) | 198 (55.8) |  |
| Undetermined or others | 13 (10.7) | 25 (7.0) |  |
| Collateral score, n (%) |  |  | **< 0.001** |
| Grade 0 | 60 (49.2) | 62 (17.5) |  |
| Grade 1 | 38 (31.1) | 153 (43.1) |  |
| Grade 2 | 24 (19.7) | 140 (39.4) |  |
| Onset to recanalization time (min) | 365 [298-448] | 352 [277-449] | 0.248 |
| Stent implantation, n (%) | 24 (19.7) | 88 (24.8) | 0.250 |
| Successful recanalization, n (%) | 99 (81.1) | 340 (95.8) | **< 0.001** |
| **Laboratory findings** |  |  |  |
| Total calcium (mmol/L) | 2.24 [2.14-2.32] | 2.22 [2.14-2.32] | 0.451 |
| Sodium (mmol/L) | 138.95 [134.97-140.42] | 139.10 [137.20-141.00] | **0.016** |
| Potassium (mmol/L) | 3.80 [3.42-4.16] | 3.80 [3.51-4.06] | 0.913 |
| Chloride (mmol/L) | 104.10 [102.00-106.45] | 104.50 [102.70-106.70] | 0.469 |
| Glucose (mmol/L) | 8.35 [6.80-10.20] | 7.00 [6.10-8.90] | **< 0.001** |
| CRP (mg/L) | 5.73 [2.01-14.29] | 3.28 [1.16-10.20] | **0.002** |
| White blood cell, × 10^9^/L | 8.75 [7.40-11.40] | 8.70 [7.20-10.70] | 0.286 |
| Neutrophil, × 10^9^/L | 7.23 [5.65-9.50] | 6.79 [5.04-8.76] | **0.025** |
| Monocyte, × 10^9^/L | 0.46 [0.31-0.60] | 0.46 [0.32-0.63] | 0.946 |
| Lymphocyte, × 10^9^/L | 0.89 [0.63-1.28] | 1.27 [0.89-1.76] | **< 0.001** |
| Hemoglobin, × 10^9^/L | 124 [113-137] | 127 [116-138] | 0.404 |
| Platelet, × 10^9^/L | 186 [135-222] | 146 [111-188] | **< 0.001** |
| PT (s) | 12.10 [11.20-13.10] | 12.00 [11.10-12.90] | 0.554 |
| TT (s) | 17.20 [15.90-18.10] | 17.20 [16.20-18.20] | 0.807 |
| APTT (s) | 27.80 [25.17-32.62] | 28.40 [26.00-32.00] | 0.350 |
| Fibrinogen (g/L) | 2.91 [2.45-3.32] | 2.94 [2.39-3.39] | 0.970 |
| INR | 1.03 [0.95-1.09] | 1.01 [0.96-1.09] | 0.458 |
| Albumin (g/L) | 39.55 [36.40-41.60] | 39.50 [37.00-42.10] | 0.441 |
| TC (mmol/L) | 4.44 [3.85-5.00] | 4.35 [3.71-5.03] | 0.593 |
| TG (mmol/L) | 1.25 [0.88-2.01] | 1.19 [0.87-1.73] | 0.179 |
| LDL-C (mmol/L) | 2.15 [1.65-2.81] | 2.18 [1.64-2.62] | 0.512 |
| HDL-C (mmol/L) | 1.31 [1.10-1.47] | 1.32 [1.10-1.52] | 0.767 |
| CALLY index | 0.66 [0.19-2.37] | 1.59 [0.53-4.24] | **< 0.001** |

Bold values indicate statistical significance.

NIHSS: National Institute of Health Stroke Scale; ASPECTS: Alberta Stroke Program Early CT Score; HMCAS: Hyperdense middle cerebral artery sign; ICA: Internal carotid artery; MCA: Middle cerebral artery; TOAST: Trail of ORG 10172 in Acute Stroke Treatment; LAA: Large-artery atherosclerosis; CRP: C-reactive protein; PT: Prothrombin time; TT: Thrombin time; APTT: Activated partial thromboplastin time; INR: International normalized ratio; TC: Total cholesterol; TG: Triglyceride; HDL-C: High-density lipoprotein cholesterol; LDL-C: Low-density lipoprotein cholesterol; CALLY index: CRP-albumin-lymphocyte index.

Supplementary Table S8. Comparisons between the CALLY index and other composite inflammatory indices using Delong tests.

|  | Hemorrhagic transformation | |  | Malignant cerebral edema | |  | Unfavorable outcome | |  | Mortality | |
| --- | --- | --- | --- | --- | --- | --- | --- | --- | --- | --- | --- |
|  | AUC value | p value |  | AUC value | p value |  | AUC value | p value |  | AUC value | p value |
| CALLY | 0.689 (0.638-0.740) | - |  | 0.637 (0.577-0.698) | - |  | 0.674 (0.626-0.722) | - |  | 0.629 (0.571-0.687) | - |
| SII | 0.680 (0.629-0.731) | 0.778 |  | 0.675 (0.619-0.730) | 0.245 |  | 0.704 (0.657-0.751) | 0.307 |  | 0.710 (0.658-0.762) | 0.013 |
| SIRI | 0.603 (0.548-0.657) | 0.007 |  | 0.585 (0.525-0.646) | 0.132 |  | 0.634 (0.584-0.684) | 0.173 |  | 0.650 (0.595-0.705) | 0.552 |
| NLR | 0.664 (0.611-0.717) | 0.422 |  | 0.627 (0.567-0.686) | 0.729 |  | 0.682 (0.634-0.730) | 0.771 |  | 0.663 (0.608-0.718) | 0.294 |
| LMR | 0.577 (0.521-0.634) | 0.001 |  | 0.573 (0.511-0.635) | 0.075 |  | 0.615 (0.564-0.665) | 0.048 |  | 0.648 (0.593-0.703) | 0.585 |
| PNI | 0.604 (0.550-0.657) | 0.006 |  | 0.580 (0.518-0.643) | 0.072 |  | 0.603 (0.552-0.653) | 0.013 |  | 0.604 (0.547-0.661) | 0.404 |
| PIV | 0.620 (0.565-0.674) | 0.036 |  | 0.624 (0.564-0.683) | 0.696 |  | 0.653 (0.604-0.702) | 0.495 |  | 0.690 (0.637-0.744) | 0.077 |

CALLY: CRP-albumin-lymphocyte index; SII: systemic immune-inflammation index; SIRI: systemic immune-inflammation response index; NLR: neutrophil-to-lymphocyte ratio; LMR: lymphocyte-to-monocyte ratio; PNI: Prognostic Nutritional Index; PIV: pan-immune-inflammation value; AUC: Area Under The Curve.

Supplementary Table S9. Baseline characteristics of patients with and without post-MT CALLY index.

|  | With post-MT CALLY index | Without post-MT CALLY index | p value |
| --- | --- | --- | --- |
| **Demongraphics** |  |  |  |
| Female, n (%) | 152 (43.3) | 60 (47.6) | 0.403 |
| Age (years) | 72 [63-78] | 71 [63-78] | 0.877 |
| Smoker, n (%) | 64 (18.2) | 29 (23.0) | 0.245 |
| Alcoholism, n (%) | 66 (18.8) | 20 (15.9) | 0.463 |
| **Medical history, n (%)** |  |  |  |
| Hypertension | 171 (48.7) | 58 (46.0) | 0.605 |
| Diabetes mellitus | 62 (17.7) | 36 (28.6) | 0.009 |
| Coronary heart disease | 42 (12.0) | 20 (15.9) | 0.263 |
| Atrial fibrillation | 188 (53.6) | 59 (46.8) | 0.194 |
| Rheumatic heart disease | 28 (8.0) | 7 (5.6) | 0.371 |
| Heart failure | 19 (5.4) | 10 (7.9) | 0.309 |
| Prior stroke | 50 (14.2) | 13 (10.3) | 0.264 |
| Antiplatelet at onset | 20 (5.7) | 4 (3.2) | 0.266 |
| Anticoagulant at onset | 33 (9.4) | 14 (11.1) | 0.581 |
| **Clinical and imaging characteristics** |  |  |  |
| Systolic pressure (mmhg) | 144 [123-162] | 149 [127-165] | 0.037 |
| Diastolic pressure (mmhg) | 83 [74-93] | 87 [77-95] | 0.011 |
| Intravenous thrombolysis, n (%) | 122 (34.8) | 45 (35.7) | 0.847 |
| Initial NIHSS score | 15 [11-19] | 15 [12-19] | 0.721 |
| Baseline ASPECTS | 8 [8-9] | 8 [7-9] | 0.012 |
| Present HMCAS, n (%) | 118 (33.6) | 49 (38.9) | 0.287 |
| Occluded vessel region, n (%) |  |  | 0.984 |
| ICA | 125 (35.6) | 45 (35.7) |  |
| MCA | 226 (64.4) | 81 (64.3) |  |
| TOAST classification, n (%) |  |  | 0.178 |
| LAA | 116 (33.0) | 53 (42.1) |  |
| Cardioembolic | 205 (58.4) | 65 (51.6) |  |
| Undetermined or others | 30 (8.5) | 8 (6.3) |  |
| Collateral score, n (%) |  |  | 0.097 |
| Grade 0 | 81 (23.1) | 41 (32.5) |  |
| Grade 1 | 143 (40.7) | 48 (38.1) |  |
| Grade 2 | 127 (36.2) | 37 (29.4) |  |
| Onset to recanalization time (min) | 355 [284-451] | 354 [280-442] | 0.602 |
| Stent implantation, n (%) | 93 (26.5) | 19 (15.1) | 0.010 |
| Successful recanalization, n (%) | 350 (99.7) | 89 (70.6) | < 0.001 |
| **Laboratory findings** |  |  |  |
| Total calcium (mmol/L) | 2.23 [2.14-2.32] | 2.22 [2.14-2.31] | 0.545 |
| Sodium (mmol/L) | 139.00 [136.90-140.80] | 139.40 [137.70-141.12] | 0.109 |
| Potassium (mmol/L) | 3.82 [3.50-4.10] | 3.80 [3.45-3.99] | 0.114 |
| Chloride (mmol/L) | 104.20 [102.30-106.40] | 105.20 [103.15-107.10] | 0.067 |
| Glucose (mmol/L) | 7.30 [6.10-9.10] | 7.45 [6.37-9.85] | 0.444 |
| CRP (mg/L) | 4.50 [1.42-12.16] | 2.65 [0.93-10.36] | 0.010 |
| White blood cell, × 10^9^/L | 8.90 [7.30-10.90] | 8.40 [7.00-10.51] | 0.115 |
| Neutrophil, × 10^9^/L | 7.15 [5.29-8.99] | 6.37 [4.77-8.73] | 0.048 |
| Monocyte, × 10^9^/L | 0.46 [0.32-0.64] | 0.47 [0.32-0.57] | 0.299 |
| Lymphocyte, × 10^9^/L | 1.16 [0.82-1.66] | 1.19 [0.82-1.60] | 0.731 |
| Hemoglobin, × 10^9^/L | 126 [115-138] | 128 [116-139] | 0.307 |
| Platelet, × 10^9^/L | 154 [114-192] | 165 [117-214] | 0.076 |
| PT (s) | 12.10 [11.20-13.00] | 11.90 [10.90-12.82] | 0.174 |
| TT (s) | 17.20 [16.10-18.20] | 17.10 [16.00-17.92] | 0.202 |
| APTT (s) | 28.70 [26.10-33.00] | 26.80 [24.87-30.35] | < 0.001 |
| Fibrinogen (g/L) | 2.98 [2.42-3.39] | 2.83 [2.38-3.39] | 0.785 |
| INR | 1.01 [0.95-1.09] | 1.03 [0.95-1.08] | 0.672 |
| Albumin (g/L) | 39.50 [36.90-41.80] | 39.50 [36.97-42.30] | 0.815 |
| TC (mmol/L) | 4.33 [3.74-5.00] | 4.39 [3.71-5.07] | 0.891 |
| TG (mmol/L) | 1.19 [0.88-1.80] | 1.20 [0.88-1.75] | 0.846 |
| LDL-C (mmol/L) | 2.16 [1.66-2.64] | 2.18 [1.48-2.75] | 0.748 |
| HDL-C (mmol/L) | 1.31 [1.08-1.50] | 1.35 [1.11-1.51] | 0.281 |
| **Clinical outcomes** |  |  |  |
| Hemorrhagic transformation | 96 (27.4) | 46 (36.5) | 0.054 |
| Malignant cerebral edema | 76 (21.7) | 33 (26.2) | 0.298 |
| Unfavorable outcome | 190 (54.1) | 70 (55.6) | 0.783 |
| Mortality | 74 (21.1) | 48 (38.1) | < 0.001 |

NIHSS: National Institute of Health Stroke Scale; ASPECTS: Alberta Stroke Program Early CT Score; HMCAS: Hyperdense middle cerebral artery sign; ICA: Internal carotid artery; MCA: Middle cerebral artery; TOAST: Trail of ORG 10172 in Acute Stroke Treatment; LAA: Large-artery atherosclerosis; CRP: C-reactive protein; PT: Prothrombin time; TT: Thrombin time; APTT: Activated partial thromboplastin time; INR: International normalized ratio; TC: Total cholesterol; TG: Triglyceride; HDL-C: High-density lipoprotein cholesterol; LDL-C: Low-density lipoprotein cholesterol; CALLY index: CRP-albumin-lymphocyte index.
